# shRNA-Based Screen Identifies Endocytic Recycling Pathway Components That Act as Genetic Modifiers of Alpha-Synuclein Aggregation, Secretion and Toxicity

**DOI:** 10.1371/journal.pgen.1005995

**Published:** 2016-04-28

**Authors:** Susana A. Gonçalves, Diana Macedo, Helena Raquel, Pedro D. Simões, Flaviano Giorgini, José S. Ramalho, Duarte C. Barral, Luís Ferreira Moita, Tiago Fleming Outeiro

**Affiliations:** 1 Instituto de Medicina Molecular, Faculdade de Medicina de Lisboa, Lisboa, Portugal; 2 CEDOC, Chronic Diseases Research Center, NOVA Medical School, Faculdade de Ciências Médicas, Universidade NOVA de Lisboa, Lisboa, Portugal; 3 Instituto de Tecnologia Química e Biológica, Universidade Nova de lisboa, Oeiras, Portugal; 4 Instituto Gulbenkian de Ciência, Oeiras, Portugal; 5 Department of Genetics, University of Leicester, Leicester, United Kingdom; 6 Department of Neurodegeneration and Restorative Research, University Medical Center Göttingen, Göttingen, Germany; 7 Max Planck Institute for Experimental Medicine, Göttingen, Germany; The University of Arizona, UNITED STATES

## Abstract

Alpha-Synuclein (aSyn) misfolding and aggregation is common in several neurodegenerative diseases, including Parkinson’s disease and dementia with Lewy bodies, which are known as synucleinopathies. Accumulating evidence suggests that secretion and cell-to-cell trafficking of pathological forms of aSyn may explain the typical patterns of disease progression. However, the molecular mechanisms controlling aSyn aggregation and spreading of pathology are still elusive. In order to obtain unbiased information about the molecular regulators of aSyn oligomerization, we performed a microscopy-based large-scale RNAi screen in living cells. Interestingly, we identified nine Rab GTPase and kinase genes that modulated aSyn aggregation, toxicity and levels. From those, Rab8b, Rab11a, Rab13 and Slp5 were able to promote the clearance of aSyn inclusions and rescue aSyn induced toxicity. Furthermore, we found that endocytic recycling and secretion of aSyn was enhanced upon Rab11a and Rab13 expression in cells accumulating aSyn inclusions. Overall, our study resulted in the identification of new molecular players involved in the aggregation, toxicity, and secretion of aSyn, opening novel avenues for our understanding of the molecular basis of synucleinopathies.

## Author Summary

Synucleinopathies are neurodegenerative diseases characterized by the abnormal accumulation of a neuronal protein called alpha-Synuclein (aSyn). The normal function of this protein in the cell remains unclear, but plays a role in synaptic function and plasticity, cell differentiation and vesicular trafficking. During disease, aSyn is believed to establish aberrant protein-protein interactions, culminating with its accumulation and aggregation and the concomitant disruption of several cellular pathways. Therefore therapeutics aimed at preventing or reversing aSyn accumulation could have potential disease-modifying effects. Here, our aim was to investigate the molecular mechanisms underlying the initial steps that lead to aSyn accumulation. For this, we performed a gene silencing screen to identify novel genetic modifiers of aSyn accumulation. We identified four genetic modifiers of aSyn, including genes involved in intracellular transport and in signal transduction pathways. In the future, the biological contextualization of the early events underlying aSyn pathology, using the modifiers identified in our study, will guide us to a deeper understanding of the role of aSyn in health and disease. In conclusion, our study constitutes a significant step forward towards the understanding of the molecular mechanisms underpinning aSyn accumulation, paving the way towards the development of novel therapeutic strategies for synucleinopathies.

## Introduction

Aggregation of alpha-Synuclein (aSyn) is associated with a group of disorders known as synucleinopathies, that include Parkinson’s Disease (PD), Dementia with Lewy Bodies and Multiple System Atrophy [1–3]. The common pathological hallmark among these disorders is the accumulation of aSyn in aggregates within neurons, nerve fibers or glial cells [4,5]. Moreover, multiplications [6] as well as point mutations (A53T, A30P, E46K, H50Q, G51D and A53E) are associated with familial forms of PD [7–13].

Recent findings suggest that aSyn can oligomerize into a tetramer under physiological conditions [14–18], although this finding remains controversial [19–21]. In pathological conditions, it is widely established that aSyn can enter an amyloid pathway of aggregation, first as soluble, oligomeric species that, ultimately, can accumulate in insoluble aggregates [22]. The role of the large protein inclusions, such as Lewy bodies (LBs), is unclear, but they may actually constitute a protective mechanism in neurons to neutralize and preclude the effects of more toxic aSyn intermediates [18,23–25].

Although the function of aSyn is still unclear, it interacts with lipid membranes [26,27] and seems to be involved in vesicle recycling and neurotransmitter release at the synapse [28,29]. Moreover, it is suggested that multimeric forms of aSyn physiologically bind to phospholipids at the synapse to chaperone SNARE-complex assembly required for neurotransmitter release, while monomeric forms are increased in disease and prone to aggregate [30–33].

Work in yeast and mammalian models suggests that aSyn-mediated cytotoxicity might be associated with alterations in vesicular trafficking, such as disruption of endoplasmic reticulum to Golgi trafficking [34,35]. This could be rescued by Rab (Ras analog in brain) GTPases, which play major roles in vesicular transport, tethering, docking and fusion [36]. Moreover, different studies have shown that dysregulation of Rab family members, such as Rab3a and Rab3b (involved in exocytosis) and Rab5 and Rab7 (involved in the endocytic pathway), are associated with aSyn-induced toxicity in dopaminergic neurons of mammalian PD models [37,38].

Together with the Braak staging hypothesis, the finding that LB pathology might have spread in the brains of PD patients transplanted with embryonic nigral cells [39–41], suggests that aSyn is able to spread in a prion-like manner in the brain. This theory has recently been supported by several studies in mouse models [42–45]. In neurons, secretion of aSyn follows a non-classical pathway [46] that is calcium-dependent and is up-regulated under stress conditions [47]. In addition, aSyn can be internalized through endocytosis or the classical clathrin-dependent pathway [48,49].

In LBs, aSyn is highly phosphorylated on Ser129, contrasting with only 4% of the total protein phosphorylated at this residue in normal brain [50,51]. This suggests that phosphorylation might interfere with the aggregation process, although it is still unclear whether phosphorylation is a trigger or a consequence of aSyn aggregation. Thus, it is critical to understand whether modulating the activity of kinases and phosphatases can interfere with aSyn aggregation and/or toxicity.

Here, we conducted an unbiased RNA interference (RNAi) screen to identify modulators of aSyn oligomerization, using the bimolecular fluorescence complementation (BiFC) assay as readout. We identified genes both encoding Rab GTPases and proteins involved in signal transduction. In addition to modifying oligomerization, the identified hits also altered aSyn toxicity and later stages of the aggregation pathway. Interestingly, we found that some of the trafficking-associated identified genes also modulated the secretion of different aSyn species. Altogether, our study brings novel insight into the molecular pathways involved in aSyn aggregation, toxicity and secretion, forming the basis for the testing of novel molecules with therapeutic potential in PD and other synucleinopathies.

## Results

### A Live-Cell shRNA Screen Identifies Modulators of aSyn Oligomerization

**Fig 1:**
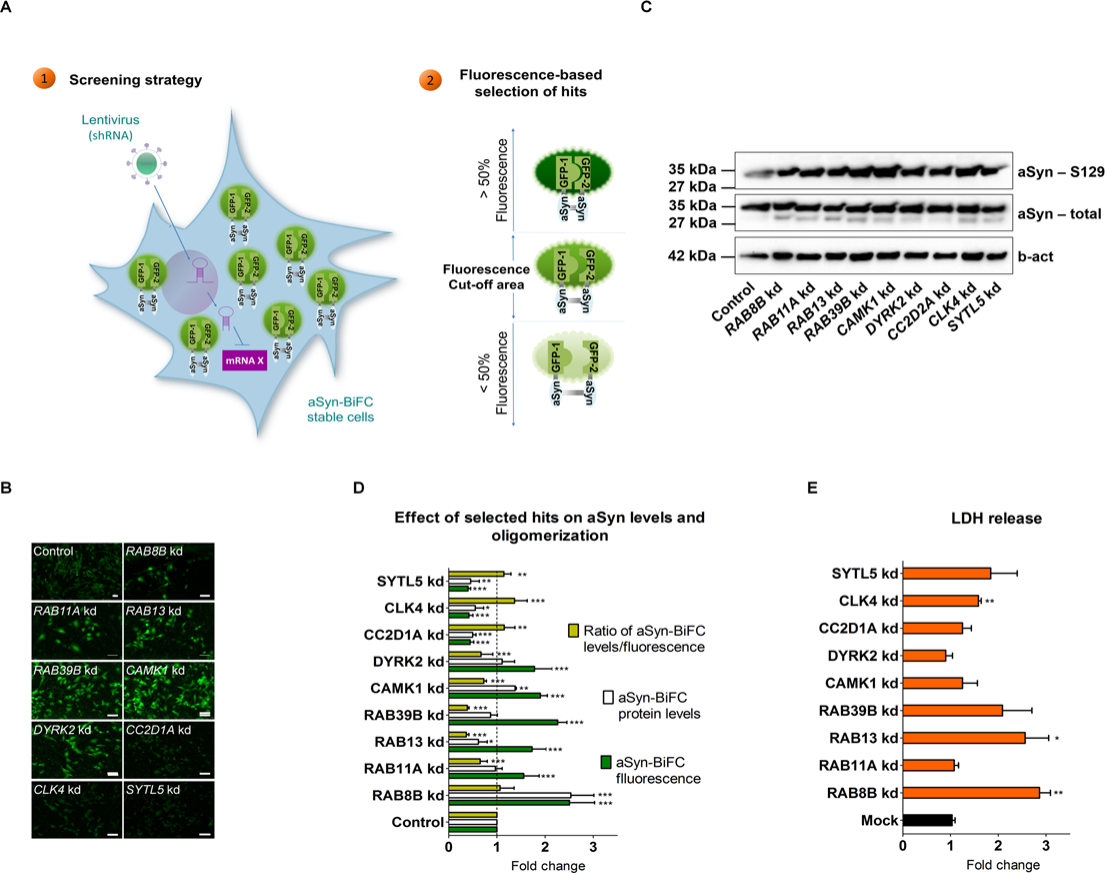
RNAi-based screen for genes that modify aSyn oligomerization in living cells. **A.** A human shRNA library targeting trafficking and phosphotransferase genes was screened using a stable cell line expressing aSyn-BiFC constructs (1). Genes modifying aSyn oligomerization by at least 50% (2) were identified using fluorescence microscopy analysis and considered for further validation. **B.** Representative live cell imaging pictures of aSyn-BiFC stable H4 cells silenced for hits that increase (*RAB8B*, *RAB11A*, *RAB13*, *RAB39B*, *CAMK1*, *DYRK2*) or decrease (*CC2D1A*, *CLK4*, *SYTL5)* aSyn oligomerization (green). A scrambled shRNA was used as control. Scale bars: 20 μm. **C.** Representative immunoblot of aSyn-BiFC cells subjected to silencing of the selected hits **D.** Relative fluorescence quantification of aSyn oligomerization (green) and quantification of aSyn protein levels (white). The ratio of protein levels and aSyn oligomerization is presented (yellow). **E.** LDH release in the media from cells with aSyn oligomers versus no aSyn (orange). Bars represent mean±95% CI (*: 0.05<p>0.01; **: 0.01<p>0.001; ***: p<0.001) and are normalized to the control of at least three independent experiments. Single comparisons between the control and experimental groups were made through Wilcoxon test. Silencing of hits was performed using at least three different shRNAs against the same gene. For simplicity, only one shRNA is shown. Results with additional shRNAs are presented in S1 Fig kd, knockdown.

In order to understand the contribution of different cellular pathways towards aSyn aggregation, we conducted an unbiased lentiviral vector-based RNAi screen in a cellular model of aSyn oligomerization, based upon a BiFC assay that we have previously described [18]. The screen comprised 1387 genes involved in trafficking and signal transduction-related pathways (S1 Table and Fig 1A).

We identified four genes encoding Rab proteins (*RAB8B*, *RAB11A*, *RAB13* and *RAB39B*) and five genes encoding kinases or signal transduction proteins (*CAMK1*, *DYRK2*, *CC2D1A*, *CLK4* and *SYTL5*) that modulated aSyn oligomerization (Fig 1B and 1D and S1A Fig). Interestingly, silencing of genes encoding kinases (*ALS2CR7* and *STK32B*), or phosphatases (*PSPH and PPP2R5E*), did not affect aSyn oligomerization but altered the subcellular distribution of the oligomers. While silencing of *ALS2CR7* or *PSPH* promoted aSyn aggregation, silencing of *STK32B* or *PPP2R5*E reduced the nuclear localization of aSyn oligomers (S2 Fig).

In the remainder of the study, we focused on the genes that modified aSyn oligomerization. Evidence of gene downregulation by the shRNAs was validated by qPCR (S1B Fig) and was confirmed by at least three different shRNAs targeting the same gene. Upon silencing of the Rab GTPase genes listed above, we observed a significant increase of aSyn-BiFC fluorescence intensity, similar to the effect of silencing *CAMK1* and *DYRK2*. Conversely, the silencing of *CC2D1A*, *CLK4* and *SYTL5* led to a significant reduction of aSyn oligomerization (Fig 1B and 1D).

To further characterize the role of the hits on aSyn oligomerization, we measured the levels of aSyn in aSyn-BiFC cells where each gene was stably silenced (Fig 1C and 1D and S1C Fig). We found that aSyn protein levels were significantly increased upon silencing of *RAB8B* or *CAMK1*. Silencing of *RAB11A*, *RAB39B* or *DYRK2* did not change the levels of aSyn, but we found a decrease upon silencing of *RAB13*, *CC2D1A*, *CLK4* and *SYTL5*. In order to correlate the levels of oligomerization with changes in the protein levels of aSyn, we compared the ratio between protein levels and fluorescence intensity (Fig 1D). The increase in aSyn oligomerization upon silencing of *RAB8B* was accompanied by an increase in levels of aSyn, suggesting the effects might be related. On the other hand, in the case of *CAMK1* silencing, the ratio of aSyn protein levels versus aSyn oligomers was <1, suggesting that the increase in oligomerization was not simply due to an increase in the levels of aSyn. Moreover, the reduced oligomerization in cells silenced for *CC2D1A*, *CLK4* or *SYTL5* might be due to reduced levels of aSyn. In contrast, the increase in aSyn oligomerization upon *RAB11A*, *RAB39B* or *DYRK2* silencing seems independent of the levels of aSyn. Interestingly, despite the observed increase in aSyn oligomerization upon *RAB13* silencing, we found a reduction in aSyn levels relative to the control. To assess whether the silencing of the candidate genes was cytotoxic, we measured the release of lactate dehydrogenase (LDH) into the media as an indicator of cell-membrane integrity. We found that silencing of *RAB8B*, *RAB13* or *CLK4* resulted in an increase in cytotoxicity in cells with aSyn oligomers compared to cells with no aSyn (Fig 1E and S1D Fig).

### Loss-of-Function of Rab Proteins Promotes Both Oligomerization and Aggregation of aSyn

**Fig 2:**
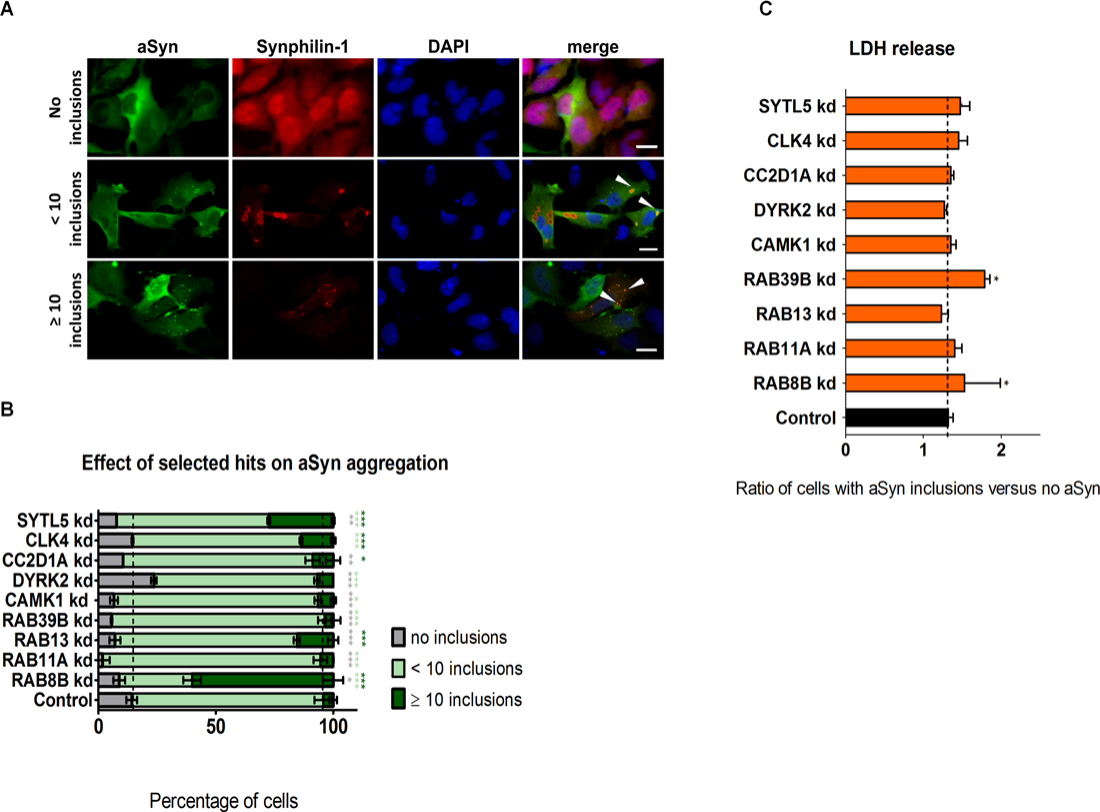
Effect of silencing of selected hits on aSyn aggregation. **A.** Stable H4 cells silenced for selected hits or with a scrambled shRNA were co-transfected with aSynT and Synphilin-1. Cells were fixed 48 h post-transfection and subjected to immunocytochemistry for aSyn (green) and for Synphilin-1 (red) followed by fluorescence microscopy. DAPI was used as a nuclear counterstain. White arrowheads point to aSyn inclusions. Scale bars: 10 μm. **B.** Percentage of cells with no inclusions (gray), less than 10 inclusions (light green) or more than 10 inclusions (dark green). **C.** Cytotoxicity (measured by LDH release in the media) from stable cells subjected to hits silencing and normalized to control (cells transduced with scrambled shRNA). The ratio represented refers to cells with aSyn inclusions versus cells with no aSyn. Bars represent mean±95% CI (*: 0.05<p>0.01; **: 0.01<p>0.001; ***: p<0.001) and are normalized to the control of at least three independent experiments. Single comparisons between the control and experimental groups were made through Wilcoxon test. kd, knockdown.

Since aSyn oligomerization precedes the formation of larger inclusions, we next asked whether the hits identified in the screen would also modulate later stages of aSyn aggregation. To test this hypothesis, we used an established model of aSyn aggregation that results in the accumulation of LB-like inclusions in H4 cells [52–54]. We co-transfected a C-terminal modified version of aSyn (aSynT) and Synphilin-1 in cells stably transduced with lentiviruses encoding shRNAs targeting each of the identified hits, and then assessed inclusion formation using immunocytochemistry and fluorescence microscopy (Fig 2A and 2B and S3A Fig). We quantified the percentage of cells according to the pattern of aSyn distribution, *i*.*e*. cells with no inclusions, cells with less than ten or cells with more than ten inclusions.

In the conditions tested, more than 80% of control cells presented less than ten intracellular inclusions, 14% did not present inclusions, and less than 4% of the cells displayed ten or less inclusions. In contrast, the silencing of all the hits except *CLK4* and *DYRK2* resulted in an increase in the percentage of cells displaying aSyn inclusions (Fig 2A and 2B and S3A Fig). Moreover, with the exception of *RAB39*, the silencing of all hits caused an increase in the percentage of cells with more than 10 inclusions. This effect was stronger upon silencing of *RAB8B* (approximately 60% of cells displaying more than ten inclusions), followed by *SYTL5* (27%) and *RAB13* (15%). Together these data suggest that the hits can also modulate later steps of the aggregation process of aSyn. To assess whether the silencing of the different hits altered cytotoxicity in the aSyn aggregation model we measured cell membrane integrity, as described above. Only the silencing of *RAB8B* and *RAB39B* resulted in an increase in cytotoxicity (Fig 2C and S3B Fig). Interestingly, the silencing of *CLK4* resulted in the accumulation of inclusions with irregular shapes and silencing of *SYTL5* resulted in the accumulation of elongated cells (S3C Fig).

In order to further evaluate whether trafficking indeed plays a role in aSyn aggregation, we silenced another traffic component involved in exocytosis (*RAB27A*). We found that silencing of *RAB27A* increased aSyn oligomerization and did not affect aSyn levels or cytotoxicity (S4 Fig and S2 Table). Furthermore, in the aggregation model, it increased the percentage of cells displaying aSyn inclusions, without affecting toxicity (S4H and S4I Fig). Thus, the effects of *RAB27A* silencing are consistent with those observed with the hits selected in our screen.

### aSyn Cell-to-Cell Trafficking Is Increased upon Silencing of *RAB8B*, *RAB13* or *SYTL5*

We and others have previously shown that aSyn can be secreted and affect multiple steps of membrane trafficking [47,55–57]. Therefore, we next investigated whether the trafficking of aSyn was affected upon silencing of four selected hits (*RAB8B*, *RAB11A*, *RAB13* or *SYTL5*) and *RAB27A*, involved in different steps of intracellular trafficking.

**Fig 3:**
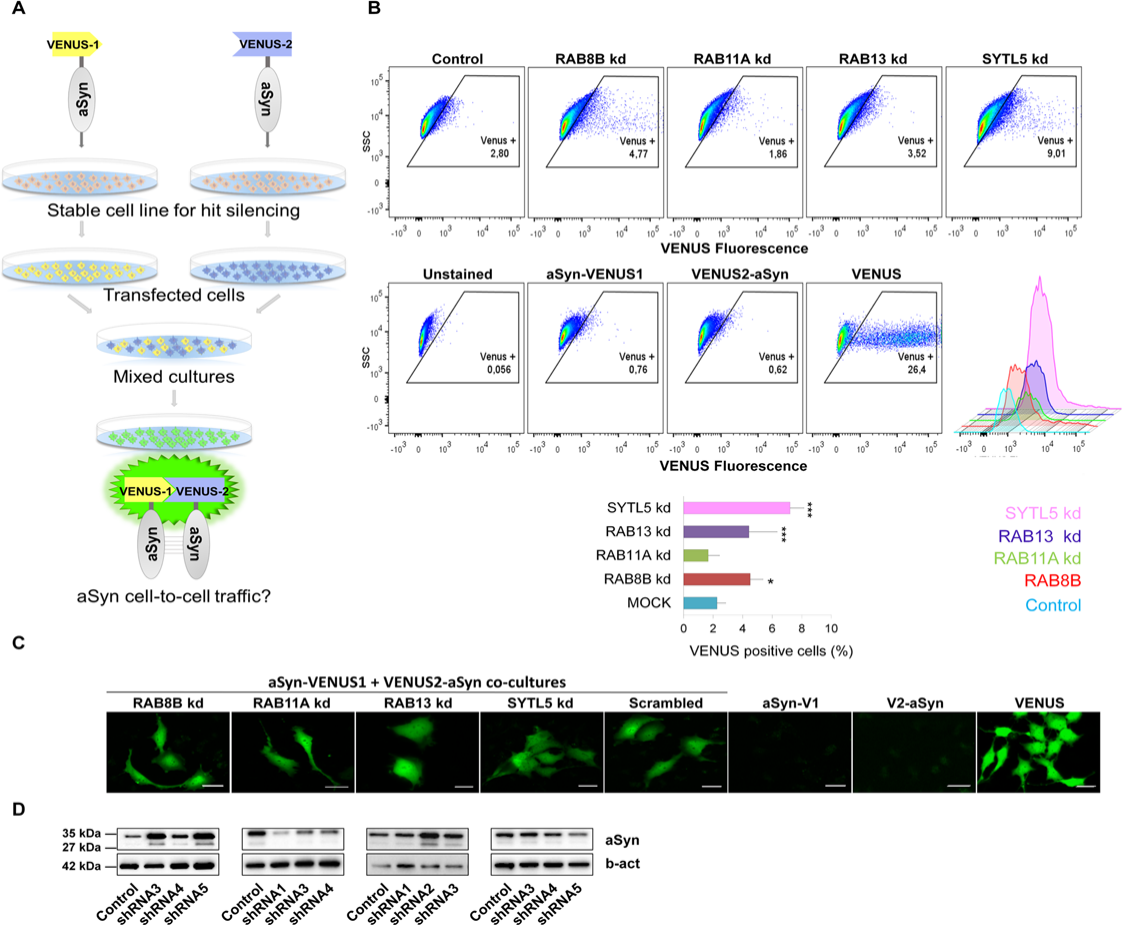
Silencing of *RAB8B*, *RAB13* or *SYTL5* increases aSyn cell-to-cell trafficking. **A.** Experimental design of aSyn cell-to-cell trafficking assay. Stable cells silenced for *RAB8B*, *RAB11A*, *RAB13*, *SYTL5* or with a scrambled shRNA were transfected separately with plasmids encoding either VENUS1-aSyn or aSyn-VENUS2. 24 h later, cells were mixed and co-cultured for 72 h. Upon release and uptake of the aSyn-VENUS fusions, reconstitution of the fluorescence signal can be detected inside cells, and the signal quantified through flow cytometry or microscopy. **B.** VENUS positive cells were monitored by flow cytometry. A representative result is shown as side scatter (SSC) versus VENUS fluorescence, with the corresponding histogram. The percentage of VENUS positive cells is indicated by the mean±95% CI of at least three independent experiments. **C.** In vivo imaging of aSyn-VENUS1 and VENUS2-aSyn mixed cells subjected to silencing of the selected hits. Scale bar: 20 μm. **D.** Immunoblotting analysis of, total aSyn and beta-actin. Bars represent mean±95% CI (*: 0.05<p>0.01; **: 0.01<p>0.001; ***: p<0.001) and are normalized to the control of at least three independent experiments. Single comparisons between the control and experimental groups were made through Wilcoxon test. kd, knockdown.

To study cell-to-cell trafficking of aSyn, we used the aSyn-BiFC system with VENUS [58,59]. Firstly, *RAB8B*, *RAB11A*, *RAB13* or *SYTL5* were silenced in H4 cells and, 24 h later, cells were transfected with either VENUS1-aSyn or aSyn-VENUS2 plasmids, separately. 24 h later, an equal number of cells transfected with VENUS1-aSyn or aSyn-VENUS2 were mixed. 72 h later, mixed cultures were analyzed by flow cytometry and microscopy for the presence of fluorescence signal, which indicates bimolecular complementation of the VENUS fluorophore and, thus, cell-to-cell trafficking of aSyn (Fig 3A). Scramble-mixed populations of VENUS1-aSyn and aSyn-VENUS2 were used to quantify cell-to-cell transfer of aSyn and used as a control to compare the effect of silencing of trafficking hits on aSyn intercellular transfer (Fig 3B). Fluorescene of cells containing a single BiFC plasmid was identical to cells without any plasmid. In the mixed population of cells that were silenced for RAB8B, RAB13 and SYTL5, we observed a significant number of fluorescent cells, indicating that transfer of aSyn between cells occurred. By knocking down RAB8B or RAB13, we observed that the number of fluorescent cells increased to 4%, double of the control situation, while SYTL5 knock down resulted in fluorescence in 7% of cells in population, more than three times the fluorescence of scrambled cells, while silencing of *RAB11A* or *RAB27A* had no effect (Fig 3B, 3C and 3D and S4E Fig).

### Endocytic Recycling of aSyn Oligomers Is Mediated by Rab11a and Rab13

**Fig 4:**
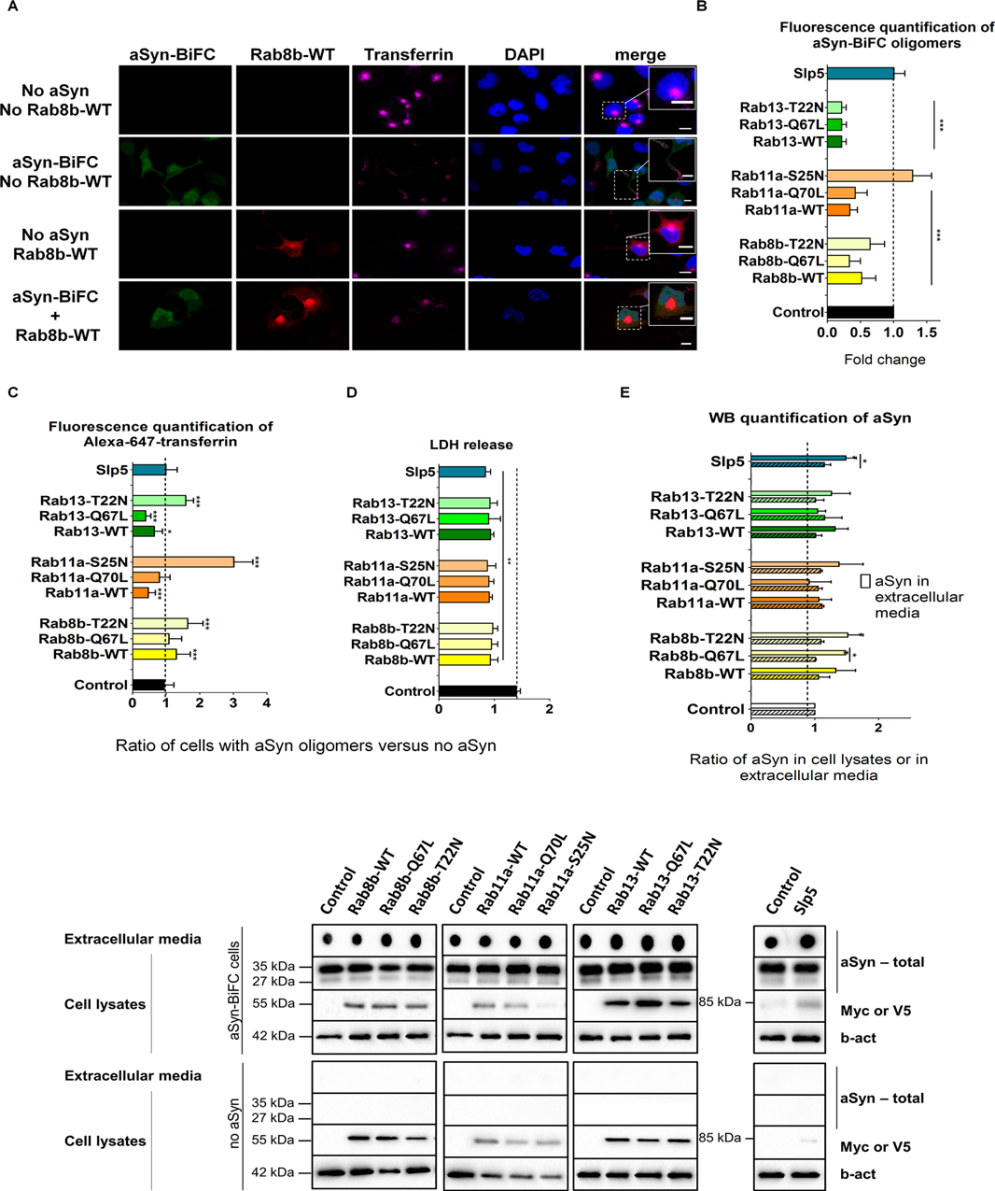
Overexpression of selected hits reduces aSyn oligomerization and modulates endocytic recycling and secretion. **A.** H4 stable cells for aSyn-BiFC (green) or H4 cells with no aSyn were transfected with constructs expressing Rab8b, Rab11a, Rab13, Slp5 (red) or empty vector. 48 h post-transfection, media with no serum was replaced in cells for 1 h. Cells were incubated with Alexa-647 human transferrin (magenta) for 30 min, prior to fixation. DAPI was used as a nuclear counterstain. Cells were subjected to confocal microscopy. For simplicity, because all expressed hits have similar subcellular locations, only wild type form of Rab8b is shown. Imaging of the remaining constructs is presented in S5, S6, S7 and S8 Figs. Squares are regions zoomed in showing transferrin localization within endocytic recycling compartment in cells with no aSyn oligomers or hit, at cells extremities in cells with aSyn oligomers and no hit, and colocalizing the hit in cells with or without aSyn. Scale bars: 10 μm. **B.** Relative aSyn-BiFC fluorescence upon hits overexpression compared to the control (empty vector transfection). **C.** Quantification of Alexa-647 transferrin intensity normalized to the control condition. The represented ratio refers to cells with aSyn oligomers versus cells with no aSyn. **D.** LDH extracellular levels were measured to assess cytotoxicity. The ratio represented refers to cells with aSyn oligomers versus cells with no aSyn. **E.** Relative quantification of aSyn intracellular total protein (stripe pattern) and extracellular conditioned media (clear pattern) for each condition. A representative immunoblot is shown. In graphs, Rab8b is represented in yellow, Rab11a in orange, Rab13 in green and Slp5 in blue. Bars represent mean±95% CI (*: 0.05<p>0.01; **: 0.01<p>0.001; ***: p<0.001) and are normalized to the control of at least three independent experiments. Single comparisons between the control and experimental groups were made through Wilcoxon test.

Next, we assessed whether overexpression of the hits selected would have the inverse effects to those observed upon silencing. For this, we expressed Rab8b, Rab11a, Rab13 and Slp5 in aSyn oligomerization model. In the case of Rab8b, Rab11a or Rab13, we compared the effects of overexpressing wild type forms, constitutively active mutants (Rab8b-Q67L, Rab11a-Q70L and Rab13-Q67L), or dominant-negative mutants (Rab8b-T22N, Rab11a-S25N and Rab13-T22N). We found that overexpression of wild type forms or the constitutively active mutants of the Rab proteins significantly reduced aSyn oligomerization, while overexpression of Slp5 had no effect (Fig 4A and 4B and S5, S6, S7 and S8 Figs). Overexpression of wild type or mutant forms of Rab13 reduced almost four times aSyn oligomerization. The dominant negative form of Rab8b, Rab8b-T22N, had a more attenuated effect on aSyn oligomerization compared to Rab8b-WT or Rab8b-Q67L. The dominant negative mutant of Rab11a did not change aSyn oligomerization.

To investigate whether the endocytic recycling pathway was altered in the presence of aSyn oligomers, we monitored the distribution of fluorescently-labeled Transferrin (Tf), which follows the endocytic recycling pathway and marks the endocytic recycling compartment (ERC). As expected, Tf accumulated in ERC in control cells without aSyn. In contrast, in cells expressing aSyn-BiFC, Tf lost the preferential accumulation in the ERC, appearing at the periphery of the cell (Fig 4A and S8B Fig). We measured the fluorescence intensity of Alexa-647-Tf and observed that the expression of wild type forms of Rab11a or Rab13 decreased the amount of the Tf within cells. In contrast, the dominant-negative forms of Rab11a and Rab13 showed a stronger Tf intracellular signal than control (Fig 4C, S6 and S7 Figs). These results indicate that overexpression of the selected Rab hits restores endocytic recycling in cells accumulating aSyn oligomers. We also found that expression of Slp5 did not alter the intracellular Tf signal, while Rab8b increased it (Fig 4C, S5A and S8A Figs).

To further determine if aSyn oligomers were secreted from cells, we measured the levels of aSyn both in the media and in cell lysates. We observed no differences in the intracellular levels of aSyn. Moreover, although secretion was slightly increased upon overexpression of all hits, only Slp5 overexpression significantly increased aSyn secretion (Fig 4E). Given that cells overexpressing each of the selected hits showed reduced cytotoxicity (Fig 4D), we concluded that the release of aSyn was not due to cell death. Overall, our results suggest that the overexpression of Rab11a and Rab13, but not Rab8b or Slp5, promotes endocytic recycling of aSyn oligomers. Also, the Slp5-mediated release of aSyn oligomers does not appear to be due to increased trafficking of aSyn via the endocytic recycling pathway.

### Overexpression of Rab11a and Rab13 Increases aSyn Secretion and Clearance of aSyn Inclusions

**Fig 5:**
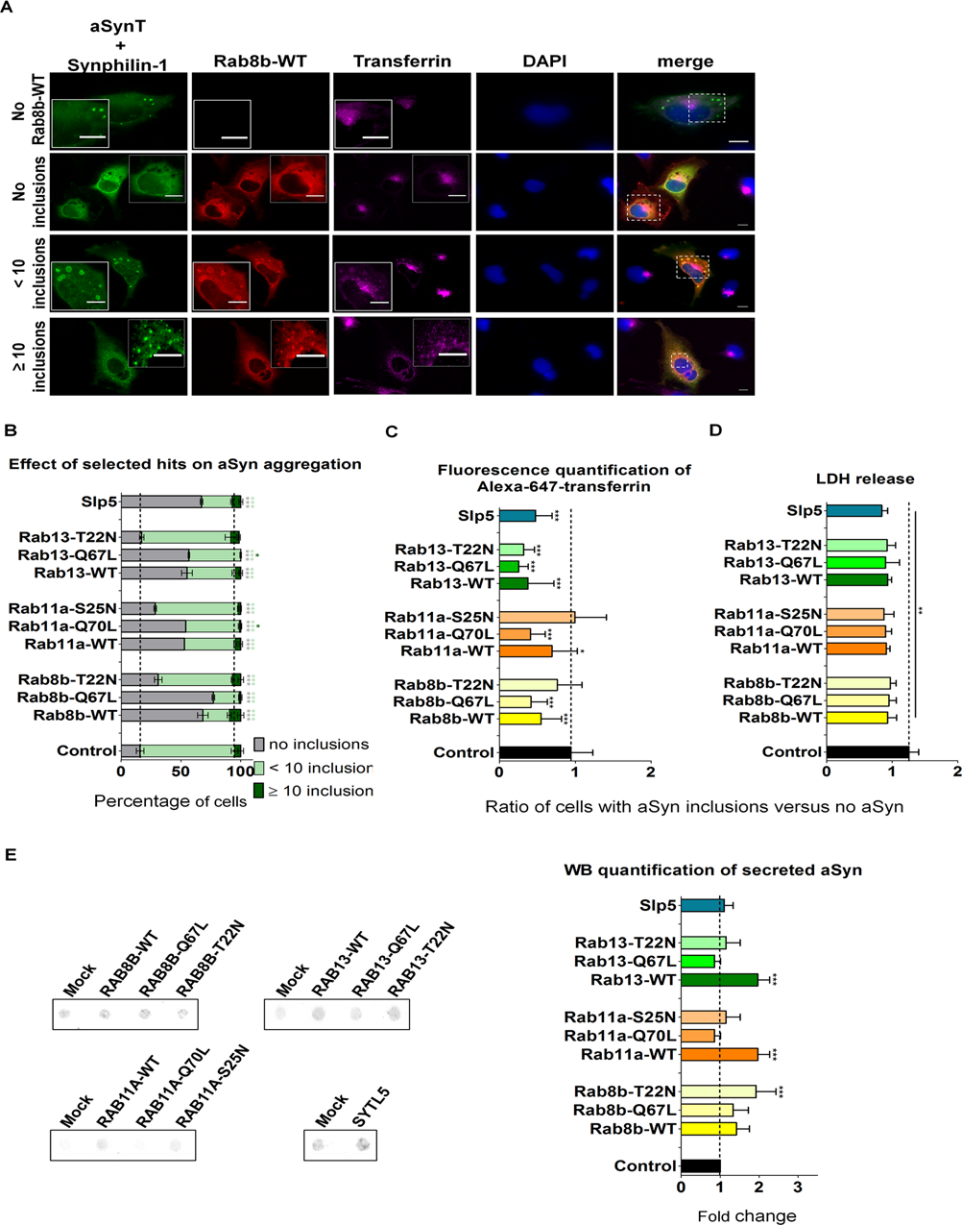
Overexpression of Rab8b, Rab11a, Rab13 and Slp5 reduces aSyn aggregation and modulates endosomal recycling and secretion. **A.** H4 cells were triple-transfected with aSynT, Synphilin-1 and constructs expressing Rab8b, Rab11a, Rab13, Slp5 (red) or empty vector. 48 h post-transfection, media with no serum was replaced in cells for 1 h. Cells were incubated with Alexa-647 human transferrin (magenta) for 30 min, prior to fixation and subjected to immunocytochemistry for aSyn (green), and followed by confocal microscopy. DAPI was used as a nuclear counterstain. Control with empty vector is shown. Amplifications within cells were made to show co-localization between aSyn, the hit and transferring within inclusions. For simplicity, only Rab8b-WT is shown. Imaging of the remaining constructs is shown in S5, S6, S7 and S8 Figs. Scale bars: 10 μm. **B.** Quantification of the number of aSyn inclusions per cell. The cells displaying aSyn inclusions were divided in: cells with no inclusions (represented in black), cells with less than 10 inclusions (in light gray) and cells with more than 10 inclusions (in dark gray). Only triple transfected cells were considered for the quantifications. **C.** Quantification of alexa-647 transferrin intensity normalized to the control condition. **D.** Cytotoxicity was measured by the LDH-release assay. The represented ratios in C and D refers to cells with aSyn inclusions versus cells with no aSyn **E.** Representative immunoblot of extracellular conditioned media from cells overexpressing the selected genes in the aSyn aggregation model, and respective quantification. In graphs, Rab8b is represented in yellow, Rab11a in orange, Rab13 in green and Slp5 in blue. Bars represent mean±95% CI (*: 0.05<p>0.01; **: 0.01<p>0.001; ***: p<0.001) and are normalized to the control of at least three independent experiments. Single comparisons between the control and experimental groups were made through Wilcoxon test.

To further explore the role of the hits identified on aSyn inclusion formation, we used the aSyn aggregation model and overexpressed Rab8b, Rab11a, Rab13 or Slp5. We found that wild type and constitutively active forms of Rab8b, Rab11a and Rab13 significantly decreased the percentage of cells with aSyn inclusions, when compared with the dominant negative forms or control. A similar effect was verified with the overexpression of Slp5 (Fig 5A and 5B). These results are consistent with those obtained upon silencing of the same genes (Fig 2B). Interestingly, in cells lacking aSyn inclusions, Rab8b, Rab11a, Rab13 and Slp5 were normally distributed in the cell, as in the control situation (Fig 4A, S5, S6, S7 and S8 Figs). However, we found that in cells with aSyn inclusions, these four proteins changed their subcellular localization and co-localized with the inclusions (Fig 5A, S5, S6, S7 and S8 Figs). Together, these results suggest that Rab8b, Rab11a, Rab13 or Slp5 can modulate aSyn aggregation and can be recruited into inclusions.

To investigate whether endocytic recycling was altered in the presence of aSyn inclusions, we monitored this process using Alexa-647-labeled Tf. Normally, Tf accumulates in the ERC. In our experiments, we found that Slp5 and wild type or constitutively active mutant forms of Rab8b, Rab11 and Rab13 decreased the intracellular fluorescence signal of Tf. In contrast, cells expressing the dominant-negative forms of these Rabs displayed similar fluorescence intensity to the controls (Fig 5C). These results indicated that recycling through the endocytic recycling pathway was compromised in cells with aSyn inclusions, as more Tf accumulated inside the cells, and that overexpression of wild type and constitutively-active forms of Rab8b, Rab11a, and Rab13, and Slp5, could rescue this defect.

To determine whether Rab8b, Rab11a, Rab13 or Slp5 played a role in aSyn secretion when this protein is aggregated, we measured the levels of aSyn in conditioned media. We found that aSyn secretion was not changed by Slp5 (Fig 5E). However, wild type forms of the Rabs increased aSyn secretion. To further confirm that the increased levels of extracellular aSyn were not due to increased cell death, we measured the release of LDH, and found that all the hits tested were protective (Fig 3D).

Altogether these results show that overexpression of Rab8b, Rab11a, Rab13 or Slp5 reduces aSyn aggregation, and that the subcellular localization of these proteins is altered in the presence of aSyn inclusions, since they all co-localize. Overexpression of these Rabs also promotes aSyn secretion, which can occur through the endocytic recycling pathway. Thus, the increased aSyn secretion upon Rab8b, Rab11a and Rab13 overexpression can explain the decrease of aSyn inclusions within the cells, as this effect is not related with an increase in cell death.

## Discussion

Increasing evidence suggests that pre-fibrillar, oligomeric forms of aSyn are the toxic species that lead to pathology [23,24,60]. The main objective of this study was to identify regulators of aSyn oligomerization, an early step of the aggregation process that precedes the formation of larger protein assemblies typically referred to as protein aggregates. To do this, we performed an RNAi screen targeting 76 membrane trafficking and 1311 phosphotransferase genes using a cell model of aSyn oligomerization. Interestingly, given the uniqueness of our approach, based on live-cell imaging of aSyn oligomers, the screen also enabled us to identify genes that did not alter aSyn oligomerization but modified the subcellular distribution of the oligomeric species.

With respect to the primary goal of the screen, we identified four genes encoding Rab proteins (*RAB8B*, *RAB11A*, *RAB13* and *RAB39B*) and five genes encoding phosphotransferase proteins (*CAMK1*, *DYRK2*, *CC2D1A*, *CLK4* and *SYTL5*) that modulated both oligomerization and aggregation (except *DYRK2*) of aSyn.

Regarding the effect of the hits on aSyn oligomerization and protein levels, we identified hits that increased both parameters, as in the case of *RAB8B* and *CAMK1*. Interestingly, silencing of *RAB8B*, but not *CAMK1*, is toxic in the presence of aSyn oligomers. The fact that *RAB8B* silencing is also toxic in the presence of aSyn inclusions suggests this is a relevant modulator at two different stages of aSyn aggregation process. Camk1 is a Calmodulin-dependent kinase that plays a role in axonal growth [61]. Until now Camk1 activity had not been associated with aSyn. However, Camk2 seems to play an essential role in the redistribution of aSyn during neurotransmitter release at the synapse [62]. Moreover, Camk2 forms a complex with aSyn and seems to regulate its oligomerization status [63]. If Camk1 and Camk2 share some functionality, this might explain the stronger downstream effect of *CAMK1* silencing, with a more pronounced effect on oligomerization rather than on the levels of aSyn. We also identified hits that decreased both aSyn oligomerization and protein levels; for example, the silencing of *CC2D1A*, *CLK4* and *SYTL5* decreased oligomerization probably because they reduce the levels of aSyn. Silencing of *CLK4*, but not of *CC2D1A* and *SYTL5*, is toxic to the cells. Thus, we can speculate that, at least for *CC2D1A* and *SYTL5*, the effects observed are not due to cytotoxicity, as membrane integrity is preserved, and these hits can be further tested as candidate therapeutic modulators in synucleinopathies. Moreover, we also found hits that had a direct effect on oligomerization without changing the levels of aSyn; silencing of *RAB11A*, *RAB39B* and *DYRK2* increased oligomerization without affecting the levels of aSyn. Silencing of *RAB39B* was toxic in the aSyn aggregation model but not in the oligomerization model. Thus, from a therapeutic perspective, hits that modify oligomerization/aggregation without altering the levels of aSyn are of great interest. Finally, we found one hit (*RAB13*) that increased oligomerization while reducing the levels of aSyn. When overexpressed, this gene was protective against toxicity, reduced oligomerization and did not alter the levels of aSyn. In total, our findings reveal an intricate connection between aSyn aggregation, toxicity and levels that will need to be further investigated in future studies.

Four out of the nine modifiers of aSyn oligomerization and aggregation were Rab small GTPases. Rab GTPases are a family of more than 60 members in humans that are master regulators of intracellular formation of vesicles, motility and release, thereby playing a key role in neuronal trafficking (reviewed in [64,65]). Rab GTPases switch between GDP-bound (inactive) and GTP-bound (active) states to regulate downstream cellular functions. It is the activation by a guanine-nucleotide exchange factor (GEF) that converts an inactive Rab into the active GTP-bound form [66]. Active Rab GTPases can bind Rab effectors, which control the spatiotemporal regulation of Rab steps within cells. Given the importance of Rab GTPases and their effectors in the regulation of membrane trafficking, several human disorders have been associated with their dysfunction, in particular diseases affecting neuronal cells (reviewed in [67]).

**Fig 6:**
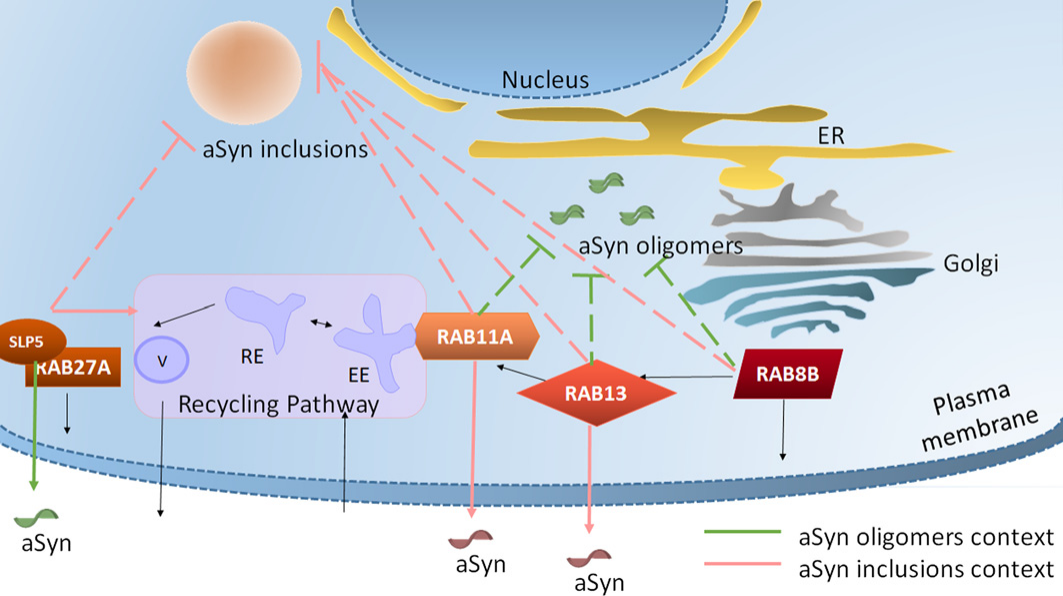
Rab8b, Rab11a, Rab13 and Slp5 are involved in different steps of cellular trafficking and modulate different aggregated species of aSyn. Rab8b is localized in cell membranes and vesicles and may be involved in polarized vesicular trafficking (endoplasmic reticulum (ER) to plasma membrane) and, specifically, in neurotransmitter release. Rab11a regulates endocytic recycling pathway and participates specifically in transferrin recycling. Rab13 plays a role in regulating membrane trafficking between trans-Golgi network, recycling endosomes (RE) and cell/tight junctions. Slp5 is localized throughout the nucleus and cytosol, and binds to phospholipidic structures. Slp5 is a Rab27a effector protein and plays a role in exocytosis. Rab8b, Rab11a and Rab13 overexpression rescues aSyn-induced toxicity and inhibits its oligomerization and aggregation. Moreover, Rab11a and Rab13 increases aSyn secretion through recycling endocytic route only when aSyn inclusions are present within cells. Although Slp5 also rescues aSyn induced toxicity when oligomerization or aggregation are the readout, it increases aSyn secretion only in a context of aSyn oligomerization. EE, early endosome; V, vesicle.

Although the hits identified fall into several different functional classes, all but *SYTL5* affect neuronal trafficking [61,68–74]. We focused on hits involved in secretion, as this process might underlie the spreading and transmission of aSyn pathology in the brain [39]. Thus, we further characterized the effect of Rab8b, Rab11a, Rab13 and Slp5 on aSyn aggregation. Rab8 is associated with actin and microtubule cell reorganization and polarized trafficking to dynamic cell surface structures [71]. Interestingly, Rab8 is able to reconstitute Golgi morphology in cellular models of PD [75] and, in addition, we recently reported that aSyn interacts with Rab8a. Moreover, we also found that Rab8 rescues the aSyn-dependent loss of dopaminergic neurons in *Drosophila* [76]. Here, we showed that silencing of Rab8b increased the accumulation of oligomeric or aggregated species of aSyn and was toxic to cells, while overexpression of Rab8b reverted those effects. Rab11a is ubiquitously expressed with preferential localization to ERC. Defective trafficking of Rab11 from the ERC has been implicated in AD, HD and PD [72,77,78]. Rab11a is involved in the process of exocytosis of aSyn via recycling endosome [78]. Silencing of Rab11a increased accumulation of oligomeric or aggregated aSyn, while overexpression of Rab11a was protective and reverted the oligomerization and aggregation of aSyn, as we previously reported in independent studies [55,79]. Rab13 mediates trafficking between the trans-Golgi network and recycling endosomes [80] and it is associated with neuronal plasticity, neurite outgrowth, cell migration and regulation of tight junctions. Interestingly, we found that Rab13 silencing was toxic to cells with aSyn oligomers but not to cells with aSyn inclusions. On the other hand, overexpression of Rab13 reduced aSyn toxicity in both cell models. We also found that Rab11a and Rab13 decrease the amount of intracellular Tf both in the models of aSyn oligomerization and aggregation. Moreover, secretion of aSyn is also differentially affected depending on the cell model, suggesting that the endocytic recycling pathway might be used to clear aSyn aggregates, possibly through secretion. Slp5 is a calcium-dependent protein that belongs to the Synaptotagmin-like protein family. Proteins from this family contain tandem C2 domains that bind phospholipids and proteins associated with the plasma membrane. Slp5 interacts with GTP-bound Rab27a, Rab3a and Rab6a, but not with Rab8 or Rab11a [81]. As vesicles an effector of Rab27a, Slp5 mediates the tethering/docking of Rab27a-positive vesicles to the plasma membrane [82]. Moreover, it can modulate the Rab27a-mediated transport of Cystic Fibrosis Transmembrane conductance Regulator (CFTR) to the membrane [83]. Slp5 can be found in the brain and in other tissues, and was shown to promote exocytosis of dense core in PC12 cells [84]. On other hand, *SYTL5* was also identified in another RNAi screen as player in chemotaxis [85], being potentially important in the generation of inflammatory responses. To the best of our knowledge, Slp5 had not been previously associated with brain disorders. Here, we showed that Slp5 silencing decreases aSyn oligomerization and increases the number of aSyn inclusions per cell. Moreover, the recycling endocytic pathway is active upon Slp5 overexpression in cells presenting aSyn inclusions, but the levels of aSyn secretion are not altered (Fig 6 and S2 Table).

Interestingly, *RAB27A* was not identified in our primary RNAi screen (S1 Table). We hypothesize that this might be due to redundancy between Rab27 isoforms and also because this GTPase has at least eleven different effectors [82] that may mask the effects of RNAi-mediated silencing. Although silencing of *RAB27A* does not affect aSyn oligomerization or secretion, it promotes aggregation (S4 Fig). This further supports the hypothesis that trafficking components are key players in aSyn homeostasis.

Remarkably, we found that overexpression of Rab8, Rab11a, Rab13 and Slp5 significantly increases the percentage of cells without inclusions to 50–75%. Although future studies will be important to further clarify the precise molecular mechanisms involved, it is possible that these proteins reduce aSyn aggregation by affecting its release. A second important observation showed that, in the remaining cells displaying aSyn inclusions, Rab8, Rab11a, Rab13 and Slp5 localized in the inclusions together with aSyn. Therefore, and as previously suggested, the sequestration of the Rabs in the inclusions may affect their function [86].

Additional studies will be necessary to clarify the relationship between the genes we identified and their cellular functions, especially those related to the endocytic recycling pathway. For example, in addition to Rab11a, Rab8 also assists the transport of Tf within cells and colocalizes with Slp1 and Slp4 [87,88]. In fact, in cells with no inclusions, transferrin (Tf) labels the endocytic recycling compartment (ERC). In cells with inclusions, the ERC location of Tf was maintained but the signal was weaker. However, if one of the selected traffic hits is overexpressed in cells with few aSyn inclusions, Tf can be seen at i) the ERC (as the traffic hit) and ii) in aSyn inclusions, co-localizing with the hit. If the number of inclusions is higher, Tf signal loses the ERC location (as the overexpressed hit) and is redistributed in inclusions. This sequential difference in Tf location reflects the possible redistribution of trafficking players and, thus, represents an alteration in the endocytic recycling machinery promoted by aSyn aggregation. This effect can synergistically be explained by a first cellular attempt to flow the excess of aSyn within the cell specifically when it is aggregated. The increase in aSyn secretion in the aSyn aggregation model (upon expression of all selected hits) suggests that endocytic recycling is being activated as less Tf signal is detected. However, when there are more inclusions in the cells there is a higher chance that the hits will be sequestered in the inclusions. aSyn is known to be secreted under physiological conditions, possibly via unconventional exocytosis, as it lacks an ER-targeting signal peptide. Although the precise mechanisms involved are still unclear, multiple secretory pathways have been described [56]. However, it was demonstrated that pathological and aggregated species of aSyn can also be secreted [89]. This suggests that misfolded and aggregated aSyn is a key agent for the propagation of PD pathology by a prion-like mechanism [90,91]. In this context, aggregation can be viewed as a protective mechanism, as it could arrest the toxic species that would otherwise be secreted. This is consistent with several observations by different groups, including our own, that protein aggregates (or at least some types of aggregates) appear to be less toxic than smaller, oligomeric species. It is possible that, after a certain threshold, the cumulative failure of cellular quality control systems, together with the secretion of aSyn, disrupts the initial cellular attempt to contain pathological aSyn species. As a result, toxic species of aSyn can spread in a prion-like manner.

Since we found that silencing of *RAB8B*, *RAB13* or *SYTL5* augmented aSyn cell-to-cell transfer (Fig 3B and 3C), these genes emerge as potential modifiers of the spreading of aSyn pathology. Transfecting independent cells with VENUS1-aSyn or aSyn-VENUS2 plasmids for 24h, and then mixing equal numbers of each cell population, enables the study of aSyn cell-to-cell transmission using the split-VENUS BiFC system. We observed a two to threefold increase in aSyn cell-to-cell transfer upon silencing of *RAB8B*, *RAB13* or *SYTL5*, while silencing of *RAB11A* or *RAB27A* had no effect when compared to scramble-infected cells. This is further supported by a stronger signal in the immunoblot of at least two of three shRNAs used to silence RAB8B, RAB13 or SYTL5. Linking these results with the oligomerization state of aSyn, silencing of both *RAB8B* and *RAB13* promoted aSyn oligomerization, probably because the balance between the entrance and exit of aSyn is increased in those cells. On the other hand, silencing of *SYTL5* decreased oligomerization and increased cell-to-cell transfer of aSyn. Recently, aSyn was shown to be secreted by exosomes [47,58,92]. Given that Slp5 is an effector protein of Rab27a involved in exosome-mediated secretion [93] and that, upon silencing, intercellular trafficking of aSyn is increased, this confirms that aSyn transmission also occurs by pathways independent of exosomes, as previously reported [55,58,94]. Silencing of *RAB11A* did not affect the cell-to-cell trafficking of aSyn (Fig 3). Hence, the increase in aSyn dimerization induced by silencing of *RAB11A* might reflect the impairment of the endocytic recycling pathway, one of the routes through which aSyn oligomers can be released [58].

In this study we showed that traffic-related modulators of aSyn oligomerization can reverse toxicity and reduce aggregation by increasing secretion of aSyn.

Altogether, the genetic screen we performed serves not only as a proof of concept for the identification of genetic modifiers of aSyn aggregation, but provides novel insight into the molecular underpinnings of PD and other synucleinopathies. Ultimately, future validation in animal models will establish which of the genes identified holds greater potential as targets for therapeutic intervention.

## Materials and Methods

### Cell Culture, Transfections, Infections and Immunocytochemistry

Human H4 neuroglial cells (HTB-148—ATCC, Manassas, VA, USA) were maintained in Opti-MEM medium supplemented with 10% of fetal bovine serum (FBS) (Life Technologies), and incubated at 37°C, 5% CO_2_. Cells were plated 24 h prior to transfection until 80% of confluence. Transfections were performed using Fugene 6 (Promega) according to the manufacturer’s instructions.

aSyn-BiFC stable cell lines were obtained by transfecting H4 cells with GN-link-aSyn and aSyn-GC constructs [18] and maintained with G418 and Hygromycin B antibiotics (both at 100 μg/ml, InvivoGen) in Opti-MEM media with 10% FBS. Green fluorescence protein (GFP) reconstitution assay was made as previously described [18] and brightest cells were viably separated using a fluorescence activated cell sorter. After growth of these selected cells, sorting and regrowth was repeated until we obtained a homogenously fluorescent aSyn-BiFC cell line.

To generate stable cell lines with hits silencing, H4 cells or aSyn-BiFC stable cells were seeded on 10 cm plates 24 h prior to infection. Cells were infected as described [95] with lentiviruses. Infected cells were selected with 5 μg/ml puromycin antibiotic (Invivogen) 48 h later and maintained with antibiotic in media.

### RNAi, High-Content Fluorescence Imaging and Analysis

#### Generation and titer of lentivirus

Lentiviral plasmids encoding shRNAs for traffic and phosphotransferase genes were obtained from the library of the RNAi Consortium (TRC). Plasmids were purified with the QiaPrep miniprep kit (QIAGEN IZASA Portugal) and transfected into HEK293T cells with a three-plasmid system to produce lentivirus with a very high titer of 10^7^ CFU/ml [95] following the standard procedures.

#### aSyn-BiFC cells infection

1.0x10^4^ stable aSyn-BiFC cells were plated in 96 well, clear bottomed, black polystyrene plates (Corning). 24 h later, the medium was carefully removed without disturbing the cells at the bottom. Cells were then infected with virus containing shRNAs for the silencing of kinases-, phosphatases- and traffic-related genes. 3–5 different shRNAs per gene were used in an arrayed format. 20 ul of virus with polyB (1:1000 final concentration) was added. The 96-well plates were then centrifuged at 2200 rpm for 90 min at 37°C. After centrifugation, medium was removed and 200 ul/well of fresh OptiMEM medium was added. 48 h post-infection, cells were washed with PBS and media (OptiMEM with no phenol red, Life technologies) was replaced. Fluorescent images were obtained from living cells using Axiovert200M microscope (Carl Zeiss MicroImaging). Over than 100 cells were acquired for each field imaging and fluorescent intensities were calculated through ImageJ software. Fluorescent screening was repeated at least three independent times and hits were selected based on the ratio of fluorescent averages. Genes which upon silencing reduced or increased the levels of GFP fluorescence by at least 50% were selected for subsequent confirmation and analysis in secondary assays. Cells were observed with 20x objective for quantification analysis and with a 63x objective for subcellular localization studies. ImageJ was used to convert the average GFP fluorescence of each cell to average pixel intensity. Values were then averaged for each condition, and statistical differences between a baseline condition and an experimental condition were calculated.

For the selected hits, production of lentiviruses was repeated as described above. 5.0x10^5^ stable aSyn-BiFC cells or H4 cells were plated in 6-well plates, and infected with 500ul of viruses with polyB (1:1000 final concentration). The 6-well plates were then centrifuged at 2200 rpm for 90 min at 37°C. After centrifugation, medium was removed and 200 ul/well of fresh OptiMEM medium was added. The plates were incubated for 48 h. Cells were selected with 5ug/ml of puromycin (final concentration).

### Real Time PCR

1.5x10^6^ aSyn-BiFC cells were plated in 10 cm plates and infected with selected hits as described [95]. 48 h post-infection, total RNA was extracted from cell lysates with Trizol reagent (Invitrogen) in accordance with the manufacturer’s instruction. 1μg of RNA was reverse transcribed into cDNA using Superscript First Strand Synthesis Kit (Invitrogen). PCR amplification was performed by using 2μl of cDNA with SYBR Green master mix (Sigma-Aldrich). Primers used for real time PCR were chosen using Primer 3, Net Primer and BLAST software to ensure specificity. RT-PCR primers here used were: for *RAB8B*, forward 5’-ATGAGGCTGGAATCCACTTG, reverse 5’-ATGAGGCTGGAATCCACTTG; for *RAB11A*, forward 5’-CATGTTCCACCAACCACTGA, reverse 5’- GTCATTCGGGACAAGTGGAT; for *RAB13*, forward 5’-CAAGACAATAACTACTGCCTACTACCG, reverse 5’-AAGCCTCATCCACATTCATACTG; for *RAB39B*, forward 5’-AGTTCCGGCTCATTGTCATC, reverse 5’- ATCTGGAGCTTGATGCGTTT; for *CAMK1*, forward 5’-AAGAGCAAGTGGAAGCAAGC, reverse 5’-AGTGAGGAGTGGTAGGGAAGC; for *DYRK2*, forward 5’-CCAGAAGTAGCAGCAGGACC, reverse 5’-CCCACTGTTGTAAGCCCATT; for *CC2D1A*, forward 5’-ATCTGGATGTCTTTGTTCGGTT, reverse 5’-TTGATGCCCTTGGTCTGG; for *CLK4*, forward 5’-GGTTGGTCTCAGCCTTGTG, reverse 5’-TGTGTTGTGGTATGGGTCCTAA; for *SYTL5*, forward 5’-AGCAAAGCCACCAAGCAC, reverse 5’-CTGAGAGTCCATCCAATCCAC; for *ACTB* (beta-actin, endogenous control), forward 5’-GGACTTCGAGCAAGAGATGG, reverse 5’-AGCACTGTGTTGGCGTACAG.

### Fluorescent-Activated Cell Sorting for Cell-to-Cell Trafficking Assay

1.5x10^6^ H4 stable cells with hit silencing (*RAB8B*, *RAB11A*, *RAB13* or *SYTL5*) per dish were plated and transfected in 10 cm dishes. 24 h later, cells were transfected cells with VENUS1-aSyn or aSyn-VENUS2 vectors independently [58]. 24 h later, 0.5x10^6^ of transfected cells with VENUS1-aSyn and aSyn-VENUS2 constructs were mixed. 72 h after, trypsin was added to each plate and neutralized with media (Opti-MEM+10% FBS). Cell suspension was centrifuged at 1100 rpm for 10 min, the supernatant aspirated and the pellet reconstituted in phosphate buffered saline (PBS). The resulting supernatant was filtered with cell strainer caps into polypropylene tubes (both from BD Biosciences). VENUS Fluorescence was measured on a BD LSRFortessa (BD Biosciences) and detected also at Axiovert200M microscope (Carl Zeiss MicroImaging).

### Overexpression Constructs

In order to generate pcDNA ENTR BP myc-mCherry-C2 mouse Rab8b, pcDNA ENTR BP V5-mCherry-C2 mouse Rab11a and pcDNA ENTR BP V5-mCherry-C2 mouse Rab13, pcDNA ENTR BP myc-mCherry-C2 or pcDNA ENTR BP V5-mCherry-C2, mammalian expression vectors were used. These mammalian expression vectors were previously generated by inserting a polylinker containing several restriction sites into pcDNA6.2GW/Em-GFP, a mammalian expression Gateway (Invitrogen) previously digested with DraI/XhoI followed by insertion of myc-mCherry-C2 or V5-mCherry-C2, previously synthetized into NheI/BamHI. Rab8b, Rab11a and Rab13 mouse coding sequence and part of 3’ UTR were produced by RT-PCR amplification using total RNA isolated from at-T20 cell line as a template, digested with EcoRI/SalI and cloned with the same restriction enzymes into the mammalian expression vectors. The primers here used were: for Rab8b, forward 5‘-AGTGAATTCATGGCGAAGACGTACGATTATCTGTTC, reverse 5‘-GACCGTCGACTCACAGGAGACTGCACCGGAAGAA; for Rab11a, forward 5‘-TGAGGAATTCATGGGCACCCGCGACGACGAGTA, reverse 5‘-AATAGTCGACCATGCTGGTTGCTGAATATGGTG; for Rab13, forward CCCGGCGCCCCCAGTGGAATTCATGGCCAAAG, reverse 5‘-GTGCGTCGACAGCCTCTCAGGACCCTAACC. Rab8b (Q67L and T22N), Rab11a (Q70L and S25N) and Rab13 (Q67L and T22N) mutants were generated by PCR mutagenesis and using the following primers: for Rab8b-Q67L, forward 5‘-GGCCTGGAAAGATTCCGAACAATTACG, reverse 5‘-CGCCGTGTCCCATATCTGAAGTTTAAT; for Rab8b-T22N, forward 5‘-GACTCCGGCGTTGGCAAGAACTGC, reverse 5‘-GCCGATGAGCAGCAGCTTGAACAGATA; for Rab11a-Q70L, forward 5‘-GGGCTGGAGCGGTACAGGGCTATAAC, reverse 5‘-TGCTGTGTCCCATATCTGTGCCTTTAT; for Rab11a-S25N, forward 5‘-GGTGTTGGAAAGAATAACCTCCTGTCT, reverse 5‘-AGAATCTCCAATAAGGACAACTTTA; for Rab13-Q67L, forward 5‘-GGCCTAGAACGATTCAAGACAATAACT, reverse 5‘-AGCCGTGTCCCACACTTGCAGTTTGAT; for Rab13-T22N, forward 5‘-TCGGGGGTGGGCAAGAATTGT, reverse 5‘-GTCCCCGATGAGCAGCAACTTGAAGAG. In order to generate pENTR V5-C2 mouse Sytl5 and pENTR GFP-C2 mouse Sytl5, Gateway mammalian expression vectors previously described were used [96]. Sytl5 mouse coding sequence was produced by RT-PCR amplification of total RNA isolated from mouse brain as template (using the primers forward 5‘-TCGAAGCTTCGGATCCATGTCTAAGAACTCAGAGTTCATC and reverse 5‘-CTAGTCGACTCAGAGCCTACATTTCGCCATGCT), digested with HindIII/SalI and cloned into pENTR GFP-C2 with the same restriction enzymes.

### aSyn-BiFC Cells Transfection with Overexpressed Hit Vectors, Transferrin Labeling and Imaging

For overexpression assays, H4 cells or aSyn-BiFC stable cells were seeded 24 h prior to transfection (on 35 mm glass bottom ibi-treated imaging dishes, ibidi GmbH) for immunocytochemistry and cell imaging or on 6 well plates for immunoblotting or cytotoxicity assays). Cells were transfected with wild type, constitutively active and dominant negative mutants of pcDNA ENTR BP myc-mCherry-C2-RAB8B (RAB8B-WT, RAB8B-Q67L, RAB8B-T22N), pcDNA ENTR BP myc-mCherry-C2-RAB11A (RAB11A-WT, RAB11A-Q70L, RAB11A-S25N), pcDNA ENTR BP V5-mCherry-C2-RAB13 (RAB13-WT, RAB13-Q67L, RAB13-T22N), pENTR V5-C2-SYLT5 constructs or empty vector (plasmids were a kind gift of Dr. José S. Ramalho, Universidade Nova de Lisboa, Portugal). 48 h post-transfection, cells were washed with PBS and incubated with media with no serum for 1 h. 50 μg/ml of Alexa-633-Tf (Life Technologies) were added for 30 min. Cells were then washed with PBS and fixed with 4% paraformaldehyde for 10 min and washed again. Immunocytochemistry was performed only for SYTL5 construct, using primary antibody (mouse anti-V5, Cell Signaling) and secondary antibody (goat anti-mouse IgG-Alexa568, Life Technologies). Nuclear staining was made using 1 μg/ml of Hoescht 33342 dye (Sigma Aldrich) for 2 min. Cells were washed and imaged in PBS. Cells were imaged using a Zeiss LSM 710 microscope with a 63× 1.4 NA oil immersion objective. Fluorescence emission was detected for Hoechst, GFP, mCherry and Far red: excitation at 405 nm (band pass 420–480), 488 nm (band pass 505–550), 561 nm (band pass 575–615), 633 nm (647–754). Pinhole was at 160 μm for all channels and 2–10% of transmission was used.

### Transferrin Labeling, Immunocytochemistry and Imaging in aSyn Aggregation Cell Model

For loss of function assays, stable H4 cells for hit silencing were seeded in 35 mm glass bottom imaging dishes (ibidi GmbH) 24 h prior to transfection and were co-transfected with aSynT and Synphilin-1-V5 as previously described [55] and subjected to immunocytochemistry 48 h later. For overexpression assays, triple transfections were with aSynT, Synphilin-1-V5 and mCherry-Rabs or GFP-Slp5 plasmids and Tf incubation was made as described for aSyn-BiFC cells. Then, cells were permeabilized with 0.5% Triton X-100 in PBS for 20 min at RT, blocked for 1 h at RT with 1% normal goat serum in 0.1% Triton X-100 in PBS, incubated with primary antibody against aSyn (mouse anti-aSyn 1:1000; BD Biosciences) and Synphilin-1-V5 (only for loss of function assays; mouse anti-V5, 1:1000, Cell Signaling) at 4°C overnight followed by secondary antibody incubation (1:1000, goat anti-mouse IgG-Alexa488 for aSynT (or igG-Alexa 568 for aSynT when co-transfected with GFP-SYTL5) and goat anti-mouse IgG-Alexa568 (Life Technologies) for Synphilin-1-V5 (only for loss of function assays), for 2 h at RT. Nuclear staining was made using 1 μg/ml of Hoechst 33342 dye (Sigma Aldrich) for 2 min. Cells were washed and imaged in PBS. Cells were then subjected to microscopy analysis using Zeiss Axiovert 200M for loss of function assays or Zeiss 710 confocal microscope for overexpression assays using the same settings used for dimerization model. The proportion of cells displaying aSyn-positive intracellular inclusions in the aSyn-positive cell population was determined by counting at least 100 cells in each condition. Moreover, for overexpression assays, Alexa-546-Tf fluorescence intensity was also determined using ImageJ.

### Immunoblotting of Intracellular Proteins

Total protein extracts were obtained 48 h post-transfection using standard procedures. Briefly, cells were washed twice in PBS and lysed in NP40 buffer (glycerol 10%, Hepes 20mM pH7.9, KCl 10mM, EDTA 1 mM, NP40 0.2%, DTT 1mM) containing protease and phosphatase inhibitors cocktail (1 tablet/10ml, Roche Diagnostics). Cell debris was spun down at 2,500 rpm for 10 min and supernatant were sonicated at 10mA for 15 s (Soniprep 150). Protein concentration was determined using the BCA protein assay (Thermo Scientific) and 20 μg of protein lysates were resolved in 12% SDS-PAGE. Resolved proteins were transferred to nitrocellulose membranes. After quick washing in TBS-T (Tris buffered saline and 0.1% Tween 20), membranes were blocked either in 5% non-fat dry milk in TBS-T or in 5% BSA in TBS-T for 1 h and then incubated with primary antibodies in 5% BSA in TBS overnight at 4°C. The primary antibodies used were mouse anti-aSyn, 1:1,000, BD Transduction; mouse anti-pS129aSyn 1:1,000 Wako Chemicals USA; mouse anti-beta actin, 1:4,000, Sigma; mouse anti-V5, 1:1,000, Cell Signaling; mouse anti-myc, 1:1,000, Santa Cruz. The membrane was then washed three times for 10 min each in TBS-T at room temperature and probed with IgG horseradish peroxidase-conjugated (HRP) anti-mouse secondary antibody (1:10,000) for 1 h at room temperature. The membrane was washed again four times for 15 min each with TBS-T and the signal was detected with an ECL chemiluminescence kit (Millipore Immobilon Western Chemiluminescent HRP Substrate).

### Immunoblotting of Extracellular aSyn

1.5x10^5^ cells were plated in 6 well plates one day prior to transfection (for overexpression assays). 48 h post-transfection or seeding (for loss-of-function assays we used stable cell lines with hits silencing), media was extracted. Using a dot-blot apparatus with a nitrocellulose membrane, 380 ul of media were loaded into wells of the dot blot templates and proteins were trapped on the membrane by vacuum. Blocking, washing and detection was made as described above, by immunoblotting the membrane.

### Cytotoxicity Assays

For aSyn cytotoxicity assay, stable H4 cells for aSyn-BiFC system or H4 cells were transduced with lentiviruses (for assays with hits loss-of-function) or transfected with overexpression vectors as described above (for overexpression assays). 48 h post-transfection or transduction, culture media was used to determine the levels of released LDH as described in the manufacturer’s instructions (Clontech Laboratories). LDH levels in the culture media were measured and ratio of toxicity between cells with aSyn dimers versus cells with no aSyn was determined.

### Statistical Analysis

Statistical significance was determined using the paired t-test with Wilcoxon matched pairs test and 95% confidence interval. Differences were considered statistically significant when p≤0.05. Analyses were performed using the Graphpad Prism 5.0 software (GraphPad Software, CA, USA).

## Supporting Information

S1 Table List of screened genes in the RNAi assay using aSyn-BiFC stable cells as readout. **A.** Human trafficking collection **B.** Human kinases / phosphatases collection.
(DOCX)

S2 Table Summary of the effect of traffic players on oligomerization and aggregation of aSyn. (DOCX)

S1 Fig Silencing of specific trafficking and kinase genes modifies aSyn oligomerization. **A.** Quantification of relative fluorescence intensity of aSyn-BiFC stable H4 cells submitted to silencing of *RAB8B*, *RAB11A*, *RAB13*, *RAB39B*, *CAMK1 DYRK2*, *CC2D1A*, *CLK4 and SYTL5*. Three different shRNAs were used per gene. **B.** mRNA levels of cells submitted to silencing of the hits normalized to control cells. **C.** Immunoblotting analysis of S129 phosphorylated aSyn, total aSyn and beta-actin. Quantification of aSyn protein levels from aSyn-BiFC cells submitted to silencing of the selected hits **D.** Cytotoxicity (measured by LDH release in media from cells with aSyn oligomers versus no aSyn) normalized to control cells. All the quantifications presented are normalized to the control cells infected with a scrambled shRNA. Bars represent mean±95% CI (*: 0.05<p>0.01; **: 0.01<p>0.001; ***: p<0.001) and are normalized to the control of at least three independent experiments. Single comparisons between the control and experimental groups were made through Wilcoxon test. kd, knockdown.
(TIF)

S2 Fig Silencing of specific phosphotransferase genes does not affect aSyn oligomerization but alters the distribution of oligomers. Upon silencing of *ALS2CR7* or *PSPH*, aSyn aggregates are seen within cells. Silencing of *STK32B* and *PPP2R5E* leads to reduced fluorescence in the nucleus. In addition, a ring of fluorescent signal surrounding the nucleus is observed. Scale bars: 20 μm.
(TIF)

S3 Fig Silencing of selected hits alters aSyn aggregation and cellular homeostasis. **A.** Quantification of the number of aSyn inclusions per cell. 3 different shRNAs per gene were used. The number of inclusions was divided in the following categories: no inclusions (gray), less than 10 inclusions (light green) and more than 10 inclusions (dark green). **B.** Cytotoxicity (measured by LDH release in media) from cells with aSyn inclusions versus no aSyn and normalized to control cells. All quantifications are normalized to the control (scrambled infected cells). Bars represent mean±95% CI (*: 0.05<p>0.01; **: 0.01<p>0.001; ***: p<0.001) and are normalized to the control of at least three independent experiments. Single comparisons between the control and experimental groups were made through Wilcoxon test. **C.** Immunohistochemistry of cells expressing aSyn and silenced for *CLK4* and *SYTL5*. Silencing of *SYTL5* in aSyn-expressing cells promote cell elongation. Upon *CLK4* depletion, aSyn inclusions adopt an amorphous shape. Scale bars: 20 μm. kd, knockdown.
(TIF)

S4 Fig Silencing of *RAB27A* alters aggregation of aSyn. **A.** Quantification of relative fluorescence intensity of aSyn-BiFC stable H4 cells submitted to silencing of *RA27A*. Three different shRNAs were tested. **B.** mRNA levels of cells submitted to silencing of the *RAB27A* normalized to control cells (cells transduced with scrambled shRNA). **C.** Immunoblotting analysis of S129 phosphorylated aSyn, total aSyn and beta-actin. Quantification of aSyn protein levels from aSyn-BiFC cells submitted to silencing of *RAB27A* **D.** Cytotoxicity (measured by LDH release in media from cells with aSyn oligomers versus no aSyn) normalized to control cells. **E.** VENUS positive cells were monitored by flow cytometry. A representative result is shown as side scatter (SSC) versus VENUS fluorescence, with the corresponding histogram. **F.** In vivo imaging of aSyn-VENUS1 and VENUS2-aSyn mixed cells subjected to silencing of *RAB27A*. Scale bar: 20 μm. **G.** Immunoblotting analysis of total aSyn and beta-actin. **H.** Percentage of cells with no inclusions (gray), less than 10 inclusions (light green) or more than 10 inclusions (dark green). **I.** Cytotoxicity (measured by LDH release in the media) from stable cells subjected to *RAB27A* silencing and normalized to control. Bars represent mean±95% CI (*: 0.05<p>0.01; **: 0.01<p>0.001; ***: p<0.001) and are normalized to the control of at least three independent experiments. Single comparisons between the control and experimental groups were made Wilcoxon test. kd, knockdown.
(TIF)

S5 Fig Overexpression of Rab8b at different steps of aSyn aggregation. H4 cells with no aSyn or stable for aSyn-BiFC (green) were transfected with Rab8b-WT,–Q67L and–T22N constructs. To promote the formation of aSyn inclusions, cells were triple-transfected with aSynT, Synphilin-1 and the same constructs referred above. 48 h post-transfection, media with no serum was replaced in cells for 1 h. Cells were incubated with Alexa-647 human transferrin (magenta) for 30 min, prior to fixation. DAPI was used as a nuclear counterstain. Only for aSyn aggregation model, cells were subjected to immunocytochemistry for aSyn (green) followed by confocal microscopy. Scale bars: 20 μm. Control cells are represented in S8B Fig.
(TIF)

S6 Fig Overexpression of Rab11a at different steps of aSyn aggregation. H4 cells with no aSyn or stable for aSyn-BiFC (green) were transfected with constructs expressing Rab11a-WT, Q70L and -S25N. To promote the formation of aSyn inclusions, cells were triple-transfected with aSynT, Synphilin-1 and the same constructs referred above. 48 h post-transfection, media with no serum was replaced in cells for 1 h. Cells were incubated with Alexa-647 human transferrin (magenta) for 30 min, prior to fixation. DAPI was used as a nuclear counterstain. Only for aSyn aggregation model, cells were subjected to immunocytochemistry for aSyn (green) followed by confocal microscopy. Scale bars: 20 μm. Control cells are represented in S8B Fig.
(TIF)

S7 Fig Overexpression of Rab13 at different steps of aSyn aggregation. H4 cells with no aSyn or stable for aSyn-BiFC (green) were transfected with constructs expressing Rab13-WT, –67L and–T22N. To promote the formation of aSyn inclusions, cells were triple-transfected with aSynT, Synphilin-1 and the same constructs referred above. 48 h post-transfection, media with no serum was replaced in cells for 1 h. Cells were incubated with Alexa-647 human transferrin (magenta) for 30 min, prior to fixation. DAPI was used as a nuclear counterstain. Only for aSyn aggregation model, cells were subjected to immunocytochemistry for aSyn (green) followed by confocal microscopy. Scale bars: 20 μm. Control cells are represented in S8B Fig.
(TIF)

S8 Fig Overexpression of SLP5 at different steps of aSyn aggregation. H4 cells with no aSyn or stable for aSyn-BiFC (green) were transfected with (**A**) SLP5 or (**B**) empty vector. To promote the formation of aSyn inclusions, cells were triple-transfected with aSynT, Synphilin-1 and the same constructs referred above. 48 h post-transfection, media with no serum was replaced in cells for 1 h. Cells were incubated with Alexa-647 human transferrin (magenta) for 30 min, prior to fixation. DAPI was used as a nuclear counterstain. Only for aSyn aggregation model, cells were subjected to immunocytochemistry for aSyn (green) followed by confocal microscopy. Scale bars: 20 μm.
(TIF)
