# Correction: shRNA-Based Screen Identifies Endocytic Recycling Pathway Components That Act as Genetic Modifiers of Alpha-Synuclein Aggregation, Secretion and Toxicity

**DOI:** 10.1371/journal.pgen.1006148

**Published:** 2016-06-23

**Authors:** The PLOS Genetics Staff

There is an error in the XML that is causing the 8^th^ author’s name, Luís Ferreira Moita, to be indexed incorrectly in PubMed. The name should be indexed as Moita LF and not Ferreira Moita L. The publisher apologizes for the error.
