# Supplementary material for: shRNA-Based Screen Identifies Endocytic Recycling Pathway Components That Act as Genetic Modifiers of Alpha-Synuclein Aggregation, Secretion and Toxicity
Source: PLoS Genet. 2016 Apr 28;12(4):e1005995. doi: 10.1371/journal.pgen.1005995 (PMC4849646; doi:10.1371/journal.pgen.1005995)
Supplement: S1 Table — A. Human trafficking collection B. Human kinases / phosphatases collection. (DOCX) [file pgen.1005995.s001.docx]

**S1 Table. List of screened genes in the RNAi assay using aSyn-BiFC stable cells as readout. (A)** Human trafficking collection (**B)** Human kinases / phosphatases collection.

| A. Human trafficking collection | | | | | | | | |
| --- | --- | --- | --- | --- | --- | --- | --- | --- |
| Gene name | | **NM_Id** | | **Gene name** | | | **NM_Id** | |
| BET1L | | NM_016526 | | **RAB24** | | | NM_130781 | |
| BNIP1 | | NM_001205 | | **RAB25** | | | NM_020387 | |
| EPIM | | NM_001980 | | **RAB26** | | | NM_014353 | |
| GOSR1 | | NM_004871 | | **RAB27A** | | | NM_004580 | |
| GOSR2 | | NM_004287 | | **RAB27B** | | | NM_004163 | |
| RAB1B | | NM_030981 | | **RAB28** | | | NM_004249 | |
| RAB2B | | NM_032846 | | **RAB30** | | | NM_014488 | |
| RAB2 | | NM_002865 | | **RAB31** | | | NM_006868 | |
| RAB3A | | NM_002866 | | **RAB32** | | | NM_006834 | |
| RAB3B | | NM_002867 | | **RAB33A** | | | NM_004794 | |
| RAB3C | | NM_138453 | | **RAB33B** | | | NM_031296 | |
| RAB3D | | NM_004283 | | **RAB34** | | | NM_031934 | |
| RAB4A | | NM_004578 | | **RAB35** | | | NM_006861 | |
| RAB4B | | NM_016154 | | **RAB36** | | | NM_004914 | |
| RAB5A | | NM_004162 | | **RAB37** | | | NM_175738 | |
| RAB5B | | NM_002868 | | **RAB38** | | | NM_022337 | |
| RAB5C | | NM_004583 | | **RAB39** | | | NM_017516 | |
| RAB6A | | NM_002869 | | **RAB39B** | | | NM_171998 | |
| RAB6B | | NM_016577 | | **RAB40B** | | | NM_006822 | |
| RAB6C | | NM_032144 | | **RAB40C** | | | NM_021168 | |
| RAB7 | | NM_004637 | | **SEC22L2** | | | NM_012430 | |
| RAB7L1 | | NM_003929 | | **SEC22L3** | | | NM_004206 | |
| RAB8A | | NM_005370 | | **SNAP29** | | | NM_004782 | |
| RAB8B | | NM_016530 | | **STX11** | | | NM_003764 | |
| RAB9A | | NM_004251 | | **STX12** | | | NM_177424 | |
| RAB9B | | NM_016370 | | **STX17** | | | NM_017919 | |
| RAB10 | | NM_016131 | | **STX1A** | | | NM_004603 | |
| RAB11A | | NM_004663 | | **STX3A** | | | NM_004177 | |
| RAB11B | | NM_004218 | | **STX4A** | | | NM_004604 | |
| RAB13 | | NM_002870 | | **STX5A** | | | NM_003164 | |
| RAB14 | | NM_016322 | | **STX6** | | | NM_005819 | |
| RAB15 | | NM_198686 | | **STX7** | | | NM_003569 | |
| RAB17 | | NM_022449 | | **STX8** | | | NM_004853 | |
| RAB18 | | NM_021252 | | **SYBL1** | | | NM_005638 | |
| RAB20 | | NM_017817 | | **VAMP3** | | | NM_004781 | |
| RAB21 | | NM_014999 | | **VTI1A** | | | NM_145206 | |
| RAB22A | | NM_020673 | | **VTI1B** | | | NM_006370 | |
| RAB23 | | NM_016277 | | **YKT6** | | | NM_006555 | |
| B. Human kinases / phosphatases collection | | | | | | | | |
| Gene name | **NM_Id** | | **Gene name** | | **NM_Id** | **Gene name** | | **NM_Id** |
| AAK1 | NM_014911 | | **ALS2CR2** | | NM_018571 | **C3orf29** | | NM_022485 |
| AATK | XM_375495 | | **ALS2CR7** | | NM_139158 | **C3orf48** | | NM_144714 |
| ABL1 | NM_005157 | | **AMHR2** | | NM_020547 | **C7orf16** | | NM_006658 |
| ABL1 | NM_007313 | | **ANKK1** | | NM_178510 | **C9orf96** | | XM_376921 |
| ABL2 | NM_005158 | | **ANP32A** | | NM_006305 | **CABC1** | | NM_020247 |
| ACACB | NM_001093 | | **APC** | | NM_000038 | **CALM1** | | NM_006888 |
| ACP1 | NM_007099 | | **APPL** | | NM_012096 | **CALM2** | | NM_001743 |
| ACP6 | NM_016361 | | **ARAF** | | NM_001654 | **CALM3** | | NM_005184 |
| ACPL2 | NM_152282 | | **ARF1** | | NM_001658 | **CAMK1** | | NM_003656 |
| ACPP | NM_001099 | | **ARHGAP29** | | NM_004815 | **CAMK1D** | | NM_020397 |
| ACPT | NM_080789 | | **ARHGEF2** | | NM_004723 | **CAMK1G** | | NM_020439 |
| ACVR1 | NM_001105 | | **ARMET** | | NM_006010 | **CAMK2A** | | NM_171825 |
| ACVR1B | NM_004302 | | **ARPP-21** | | NM_198399 | **CAMK2B** | | NM_001220 |
| ACVR1B | NM_020328 | | **ATM** | | NM_000051 | **CAMK2D** | | NM_001221 |
| ACVR1C | NM_145259 | | **ATP6V0E2L** | | XM_088142 | **CAMK2G** | | NM_001222 |
| ACVR2A | NM_001616 | | **ATPBD3** | | NM_145232 | **CAMK2N1** | | NM_018584 |
| ACVR2B | NM_001106 | | **ATR** | | NM_001184 | **CAMK4** | | NM_001744 |
| ADAM2 | NM_001464 | | **AURKA** | | NM_003600 | **CAMKK1** | | NM_172207 |
| ADCK1 | NM_020421 | | **AURKAIP1** | | NM_017900 | **CAMKK2** | | NM_153499 |
| ADCK2 | NM_052853 | | **AURKB** | | NM_004217 | **CAMKV** | | NM_024046 |
| ADCK5 | NM_174922 | | **AURKC** | | NM_003160 | **CARKL** | | NM_013276 |
| ADK | NM_001123 | | **AXL** | | NM_001699 | **CASK** | | NM_003688 |
| ADPGK | NM_031284 | | **AXL** | | NM_021913 | **CBL** | | NM_005188 |
| ADRBK1 | NM_001619 | | **BCKDK** | | NM_005881 | **CC2D1A** | | NM_017721 |
| ADRBK2 | NM_005160 | | **BCL2** | | NM_000633 | **CCL2** | | NM_002982 |
| AGTR2 | NM_000686 | | **BCL2L11** | | NM_138621 | **CCNB3** | | NM_033670 |
| AK1 | NM_000476 | | **BCR** | | NM_004327 | **CCND1** | | NM_053056 |
| AK2 | NM_001625 | | **BLK** | | NM_001715 | **CCR2** | | NM_000648 |
| AK3 | NM_016282 | | **BMP2K** | | NM_017593 | **Ccr2** | | NM_009915 |
| AK3L1 | NM_013410 | | **BMP2KL** | | XM_293293 | **CCRK** | | NM_178432 |
| AK5 | NM_012093 | | **BMPR1A** | | NM_004329 | **CCRN4L** | | NM_012118 |
| AK7 | NM_152327 | | **BMPR1B** | | NM_001203 | **CD40** | | NM_001250 |
| AKAP3 | NM_006422 | | **BMPR2** | | NM_001204 | **CDC14A** | | NM_003672 |
| AKAP4 | NM_003886 | | **BMX** | | NM_001721 | **CDC14C** | | NM_152627 |
| AKAP5 | NM_004857 | | **BPNT1** | | NM_006085 | **CDC2** | | NM_001786 |
| AKAP6 | NM_004274 | | **BRAF** | | NM_004333 | **CDC25A** | | NM_001789 |
| AKAP7 | NM_004842 | | **BRCA1** | | NM_007294 | **CDC25B** | | NM_004358 |
| AKAP8 | NM_005858 | | **BRCA2** | | NM_000059 | **CDC25C** | | NM_001790 |
| AKAP8L | NM_014371 | | **BRD2** | | NM_005104 | **CDC2L1** | | NM_001787 |
| AKAP9 | NM_005751 | | **BRD3** | | NM_007371 | **CDC2L2** | | NM_024011 |
| AKAP10 | NM_007202 | | **BRD4** | | NM_058243 | **CDC2L5** | | NM_003718 |
| AKAP11 | NM_016248 | | **BRDT** | | NM_001726 | **CDC2L6** | | NM_015076 |
| AKAP12 | NM_005100 | | **BRSK1** | | NM_032430 | **CDC42BPA** | | NM_014826 |
| AKAP13 | NM_006738 | | **BRSK2** | | NM_003957 | **CDC42BPA** | | NM_003607 |
| AKAP14 | NM_178813 | | **BTK** | | NM_000061 | **CDC42BPB** | | NM_006035 |
| AKT1 | NM_005163 | | **BUB1** | | NM_004336 | **CDC42BPG** | | XM_290516 |
| AKT2 | NM_001626 | | **BUB1B** | | NM_001211 | **CDC42SE2** | | NM_020240 |
| AKT3 | NM_005465 | | **C11orf17** | | NM_020642 | **CDC7** | | NM_003503 |
| ALK | NM_004304 | | **C14orf41** | | XM_495996 | **CDH1** | | NM_004360 |
| ALPK1 | NM_025144 | | **C15orf42** | | NM_152259 | **CDK10** | | NM_003674 |
| ALPK2 | NM_052947 | | **C17orf51** | | XM_378661 | **CDK10** | | NM_052987 |
| ALPK3 | NM_020778 | | **C17orf75** | | NM_022344 | **CDK2** | | NM_001798 |
| CDK2 | NM_052827 | | **CSK** | | NM_004383 | **DOK1** | | NM_001381 |
| CDK4 | NM_000075 | | **CSMD1** | | NM_033225 | **DTYMK** | | NM_012145 |
| CDK5 | NM_004935 | | **CSNK1A1** | | NM_001892 | **DULLARD** | | NM_015343 |
| CDK5R1 | NM_003885 | | **CSNK1A1L** | | NM_145203 | **DUSP1** | | NM_004417 |
| CDK6 | NM_001259 | | **CSNK1D** | | NM_001893 | **DUSP3** | | NM_004090 |
| CDK7 | NM_001799 | | **CSNK1D** | | NM_139062 | **DUSP4** | | NM_001394 |
| CDK8 | NM_001260 | | **CSNK1E** | | NM_001894 | **DUSP5** | | NM_004419 |
| CDK9 | NM_001261 | | **CSNK1E** | | NM_152221 | **DUSP6** | | NM_001946 |
| CDKL1 | NM_004196 | | **CSNK1G2** | | NM_001319 | **DUSP8** | | NM_004420 |
| CDKL2 | NM_003948 | | **CSNK1G3** | | NM_004384 | **DUSP9** | | NM_001395 |
| CDKL3 | NM_016508 | | **CSNK2A1** | | NM_001895 | **DUSP10** | | NM_007207 |
| CDKL4 | XM_293029 | | **CSNK2A1** | | NM_177559 | **DUSP11** | | NM_003584 |
| CDKL5 | NM_003159 | | **CSNK2A2** | | NM_001896 | **DUSP12** | | NM_007240 |
| CDKN1A | NM_000389 | | **CTDP1** | | NM_004715 | **DUSP13** | | NM_016364 |
| CDKN1B | NM_004064 | | **CTDSP2** | | NM_005730 | **DUSP14** | | NM_007026 |
| CDKN1C | NM_000076 | | **CYLD** | | NM_015247 | **DUSP15** | | NM_080611 |
| CDKN2A | NM_058197 | | **DAB2IP** | | NM_032552 | **DUSP18** | | NM_152511 |
| CDKN2C | NM_001262 | | **DAPK1** | | NM_004938 | **DUSP19** | | NM_080876 |
| CERK | NM_182661 | | **DAPK2** | | NM_014326 | **DUSP21** | | NM_022076 |
| CERKL | NM_201548 | | **DAPK3** | | NM_001348 | **DUSP22** | | NM_020185 |
| CHEK1 | NM_001274 | | **DAPP1** | | NM_014395 | **DUSP26** | | NM_024025 |
| CHEK2 | NM_007194 | | **DBF4** | | NM_006716 | **DUSP27** | | XM_043739 |
| CHKA | NM_001277 | | **DCAMKL1** | | NM_004734 | **DVL1** | | NM_004421 |
| CHKB | NM_005198 | | **DCAMKL2** | | NM_152619 | **DVL2** | | NM_004422 |
| CHUK | NM_001278 | | **DCAMKL3** | | XM_047355 | **DYRK1A** | | NM_001396 |
| CIB2 | NM_006383 | | **DCC** | | NM_005215 | **DYRK1B** | | NM_004714 |
| CIB3 | NM_054113 | | **DCK** | | NM_000788 | **DYRK2** | | NM_003583 |
| CIB4 | XM_059399 | | **DDR1** | | NM_001954 | **DYRK3** | | NM_003582 |
| CILP | NM_003613 | | **DDR2** | | NM_006182 | **DYRK4** | | NM_003845 |
| CINP | NM_032630 | | **DGKA** | | NM_001345 | **DYSF** | | NM_003494 |
| CIT | NM_007174 | | **DGKB** | | NM_004080 | **E2F1** | | NM_005225 |
| CKB | NM_001823 | | **DGKD** | | NM_003648 | **EEF2K** | | NM_013302 |
| CKM | NM_001824 | | **DGKE** | | NM_003647 | **EGFR** | | NM_005228 |
| CKMT1B | NM_020990 | | **DGKG** | | NM_001346 | **EGLN1** | | NM_022051 |
| CKMT2 | NM_001825 | | **DGKH** | | NM_152910 | **EGLN3** | | NM_022073 |
| CKS1B | NM_001826 | | **DGKI** | | NM_004717 | **EIF2AK1** | | NM_014413 |
| CKS2 | NM_001827 | | **DGKK** | | XM_066534 | **EIF2AK2** | | NM_002759 |
| CLK1 | NM_004071 | | **DGKQ** | | NM_001347 | **EIF2AK3** | | NM_004836 |
| CLK2 | NM_003993 | | **DGKZ** | | NM_003646 | **ELAC2** | | NM_018127 |
| CLK3 | NM_003992 | | **DGUOK** | | NM_001929 | **ELAVL4** | | NM_021952 |
| CLK4 | NM_020666 | | **DHH** | | NM_021044 | **ENDOG** | | NM_004435 |
| CMPK | NM_016308 | | **DKC1** | | NM_001363 | **ENPP1** | | NM_006208 |
| CNKSR1 | NM_006314 | | **DKFZp686K16132** | | XM_371497 | **ENPP6** | | NM_153343 |
| CNKSR3 | NM_173515 | | **DKFZp761P0423** | | XM_291277 | **ENPP7** | | NM_178543 |
| CNP | NM_033133 | | **DLEC1** | | NM_005106 | **EP300** | | NM_001429 |
| COL3A1 | NM_000090 | | **DLG1** | | NM_004087 | **EPB41L4A** | | NM_022140 |
| COL4A3BP | NM_005713 | | **DLG2** | | NM_001364 | **EPHA1** | | NM_005232 |
| CPNE1 | NM_152928 | | **DLG4** | | NM_001365 | **EPHA2** | | NM_004431 |
| CPNE2 | NM_152727 | | **DMBT1** | | NM_004406 | **EPHA3** | | NM_005233 |
| CPNE3 | NM_003909 | | **DMPK** | | NM_004409 | **EPHA4** | | NM_004438 |
| CPT2 | NM_000098 | | **DNA2L** | | XM_166103 | **EPHA5** | | NM_004439 |
| CRKL | NM_005207 | | **DNAJC6** | | NM_014787 | **EPHA6** | | NM_173655 |
| CRKRS | NM_016507 | | **DOCK2** | | NM_004946 | **EPHA6** | | XM_114973 |
| CSF1R | NM_005211 | | **DOCK4** | | NM_014705 | **EPHA7** | | NM_004440 |
| EPHA8 | NM_020526 | | **FRAP1** | | NM_004958 | **HECW1** | | NM_015052 |
| EPHA10 | NM_173641 | | **FRK** | | NM_002031 | **HGF** | | NM_000601 |
| EPHB1 | NM_004441 | | **FRMD1** | | NM_024919 | **HGS** | | NM_004712 |
| EPHB2 | NM_004442 | | **FRMPD2** | | NM_152428 | **HINT3** | | NM_138571 |
| EPHB3 | NM_004443 | | **FUK** | | NM_145059 | **HIPK2** | | NM_022740 |
| EPHB4 | NM_004444 | | **FUS** | | NM_004960 | **HIPK3** | | NM_005734 |
| EPHB6 | NM_004445 | | **FXN** | | NM_000144 | **HIPK4** | | NM_144685 |
| EPM2A | NM_005670 | | **FYN** | | NM_002037 | **HK1** | | NM_033498 |
| ERBB2 | NM_004448 | | **Gabra1** | | NM_010250 | **HK2** | | NM_000189 |
| ERBB3 | NM_001982 | | **Gabra2** | | NM_008066 | **HK3** | | NM_002115 |
| ERBB4 | NM_005235 | | **Gabra3** | | NM_008067 | **HNRPA2B1** | | NM_002137 |
| EREG | NM_001432 | | **Gabra5** | | NM_176942 | **HRAS** | | NM_176795 |
| ERN1 | NM_001433 | | **GAK** | | NM_005255 | **HRAS** | | NM_005343 |
| ESR1 | NM_000125 | | **GALK1** | | NM_000154 | **HRASLS** | | NM_020386 |
| ETNK1 | NM_018638 | | **GALK2** | | NM_002044 | **HSP90AA1** | | NM_005348 |
| ETNK2 | NM_018208 | | **GAPVD1** | | XM_044196 | **HSPA5** | | NM_005347 |
| EVI1 | NM_005241 | | **GBL** | | NM_022372 | **HSPB8** | | NM_014365 |
| EXO1 | NM_130398 | | **GCK** | | NM_033507 | **HUNK** | | NM_014586 |
| EXOSC10 | NM_002685 | | **GCKR** | | NM_001486 | **IBTK** | | XM_371835 |
| EXT1 | NM_000127 | | **GEFT** | | NM_133483 | **ICK** | | NM_014920 |
| EXT2 | NM_000401 | | **GGTL3** | | NM_178025 | **IGBP1** | | NM_001551 |
| EZH1 | NM_001991 | | **GK** | | NM_000167 | **IGF1** | | NM_000618 |
| EZH2 | NM_004456 | | **GK2** | | NM_033214 | **IGF1R** | | NM_000875 |
| FAM62A | NM_015292 | | **GKAP1** | | NM_025211 | **IHH** | | XM_050846 |
| FAS | NM_000043 | | **GLI2** | | NM_030379 | **IHPK2** | | NM_016291 |
| FASN | NM_004104 | | **GMFB** | | NM_004124 | **IHPK3** | | NM_054111 |
| FASTK | NM_006712 | | **GMFG** | | NM_004877 | **IKBKAP** | | NM_003640 |
| FCRL2 | NM_030764 | | **GMIP** | | NM_016573 | **IKBKE** | | NM_014002 |
| FER | NM_005246 | | **GNB2L1** | | NM_006098 | **ILK** | | NM_004517 |
| FER1L3 | NM_013451 | | **GNE** | | NM_005476 | **ILKAP** | | NM_030768 |
| FES | NM_002005 | | **GPR109A** | | NM_177551 | **ILVBL** | | NM_176826 |
| FGFR1 | NM_000604 | | **Gpr109a** | | NM_030701 | **INPP4A** | | NM_001566 |
| FGFR2 | NM_000141 | | **Gpr12** | | NM_008151 | **INPP4B** | | NM_003866 |
| FGFR3 | NM_000142 | | **GPSM2** | | NM_013296 | **INPP5D** | | NM_005541 |
| FGFR4 | NM_002011 | | **GRK1** | | NM_002929 | **INPP5E** | | NM_019892 |
| FGR | NM_005248 | | **GRK4** | | NM_005307 | **INPP5F** | | NM_014937 |
| FIGN | NM_018086 | | **GRK5** | | NM_005308 | **INPPL1** | | NM_001567 |
| FLJ21438 | XM_029084 | | **GRK6** | | NM_002082 | **INSR** | | NM_000208 |
| FLJ23356 | NM_032237 | | **GRK7** | | NM_139209 | **INSRR** | | NM_014215 |
| FLJ25006 | NM_144610 | | **GSC** | | NM_173849 | **IPMK** | | NM_152230 |
| FLJ30092 | XM_497354 | | **GSG2** | | NM_031965 | **IRAK1** | | NM_001569 |
| FLJ30698 | XM_375602 | | **GSK3A** | | NM_019884 | **IRAK2** | | NM_001570 |
| FLJ32658 | NM_144688 | | **GSK3B** | | NM_002093 | **IRAK3** | | NM_007199 |
| FLJ40125 | NM_178494 | | **GTF2H1** | | NM_005316 | **IRAK4** | | NM_016123 |
| FLJ40852 | NM_173677 | | **GUCY2C** | | NM_004963 | **IRS1** | | NM_005544 |
| FLT1 | NM_002019 | | **GUCY2F** | | NM_001522 | **ITCH** | | NM_031483 |
| FLT3 | NM_004119 | | **GUK1** | | NM_000858 | **ITGAV** | | NM_002210 |
| FLT3LG | NM_001459 | | **GZMA** | | NM_006144 | **ITGB3** | | NM_000212 |
| FLT4 | NM_002020 | | **GZMB** | | NM_004131 | **ITK** | | NM_005546 |
| FN3K | NM_022158 | | **GZMH** | | NM_033423 | **ITPK1** | | NM_014216 |
| FN3KRP | NM_024619 | | **GZMK** | | NM_002104 | **ITPKA** | | NM_002220 |
| FNDC3B | NM_022763 | | **GZMM** | | NM_005317 | **ITPKB** | | NM_002221 |
| FOXO1A | NM_002015 | | **HABP2** | | NM_004132 | **ITPKC** | | NM_025194 |
| FOXO3A | NM_001455 | | **HCK** | | NM_002110 | **ITSN1** | | NM_003024 |
| ITSN2 | NM_019595 | | **LOC440091** | | XM_495916 | **MAP4K5** | | NM_006575 |
| JAK1 | NM_002227 | | **LOC440345** | | XM_496125 | **MAPK1** | | NM_138957 |
| JAK2 | NM_004972 | | **LOC440354** | | XM_496137 | **MAPK3** | | NM_002746 |
| JAK3 | NM_000215 | | **LOC440388** | | XM_496170 | **MAPK4** | | NM_002747 |
| JUN | NM_002228 | | **LOC440820** | | XM_496519 | **MAPK6** | | NM_002748 |
| KALRN | NM_007064 | | **LOC441655** | | XM_497366 | **MAPK7** | | NM_139034 |
| KDR | NM_002253 | | **LOC441759** | | XM_497498 | **MAPK7** | | NM_139032 |
| KHK | NM_000221 | | **LOC441812** | | XM_497579 | **MAPK8** | | NM_139049 |
| KIAA0226 | XM_032901 | | **LOC441868** | | XM_497647 | **MAPK8IP1** | | NM_005456 |
| KIAA0999 | NM_025164 | | **LOC442075** | | XM_497910 | **MAPK8IP2** | | NM_012324 |
| KIAA1303 | NM_020761 | | **LOC442558** | | XM_499301 | **MAPK8IP3** | | NM_015133 |
| KIAA1446 | NM_020836 | | **LOC644379** | | XM_372273 | **MAPK9** | | NM_139069 |
| KIAA1639 | XM_290923 | | **LOC644644** | | XM_372274 | **MAPK9** | | NM_002752 |
| KIAA1706 | NM_030636 | | **LOC647208** | | XM_496155 | **MAPK10** | | NM_002753 |
| KIAA1804 | NM_032435 | | **LOC91461** | | XM_038576 | **MAPK10** | | NM_138982 |
| KIAA2002 | XM_370878 | | **LRPPRC** | | NM_133259 | **MAPK11** | | NM_002751 |
| KIDINS220 | XM_291015 | | **LRRK1** | | NM_024652 | **MAPK12** | | NM_002969 |
| KIT | NM_000222 | | **LRRK2** | | XM_058513 | **MAPK13** | | NM_002754 |
| KLHL23 | NM_144711 | | **LTK** | | NM_002344 | **MAPK14** | | NM_001315 |
| KRAS | NM_033360 | | **LYK5** | | NM_153335 | **MAPK14** | | NM_139012 |
| KRAS | NM_004985 | | **LYN** | | NM_002350 | **MAPK15** | | NM_139021 |
| KSR1 | XM_290793 | | **MADD** | | NM_003682 | **MAPKAP1** | | NM_024117 |
| KSR2 | NM_173598 | | **MAGI3** | | NM_020965 | **MAPKAPK2** | | NM_032960 |
| LATS1 | NM_004690 | | **MAK** | | NM_005906 | **MAPKAPK3** | | NM_004635 |
| LATS2 | NM_014572 | | **MAMDC1** | | NM_182830 | **MAPKAPK5** | | NM_003668 |
| LCK | NM_005356 | | **MAMDC2** | | NM_153267 | **MAPKBP1** | | XM_031706 |
| LIG4 | NM_002312 | | **MAP2K1** | | NM_002755 | **MARK1** | | NM_018650 |
| LIMK1 | NM_002314 | | **MAP2K1IP1** | | NM_021970 | **MARK2** | | NM_004954 |
| LIMK2 | NM_016733 | | **MAP2K2** | | NM_030662 | **MARK3** | | NM_002376 |
| LMTK3 | XM_055866 | | **MAP2K3** | | NM_002756 | **MASA** | | NM_021204 |
| LOC283155 | XM_208545 | | **MAP2K4** | | NM_003010 | **MAST1** | | NM_014975 |
| LOC283871 | XM_208887 | | **MAP2K5** | | NM_145162 | **MAST2** | | NM_015112 |
| LOC375133 | NM_199345 | | **MAP2K6** | | NM_002758 | **MAST3** | | XM_038150 |
| LOC375449 | NM_198828 | | **MAP2K7** | | NM_005043 | **MAST4** | | XM_291141 |
| LOC387870 | XM_291991 | | **MAP3K1** | | XM_042066 | **MASTL** | | NM_032844 |
| LOC387927 | XM_370726 | | **MAP3K2** | | NM_006609 | **MATK** | | NM_002378 |
| LOC388259 | XM_370975 | | **MAP3K3** | | NM_002401 | **MAX** | | NM_002382 |
| LOC389069 | XM_371588 | | **MAP3K4** | | NM_005922 | **MBIP** | | NM_016586 |
| LOC389772 | XM_372128 | | **MAP3K5** | | NM_005923 | **MCTP1** | | NM_024717 |
| LOC389873 | XM_372233 | | **MAP3K6** | | NM_004672 | **MCTP2** | | NM_018349 |
| LOC390641 | XM_497469 | | **MAP3K7** | | NM_145332 | **MELK** | | NM_014791 |
| LOC390705 | XM_372626 | | **MAP3K7IP1** | | NM_006116 | **MEN1** | | NM_000244 |
| LOC390877 | XM_372705 | | **MAP3K8** | | NM_005204 | **MERTK** | | NM_006343 |
| LOC390975 | XM_372749 | | **MAP3K9** | | XM_027237 | **MET** | | NM_000245 |
| LOC391025 | XM_372775 | | **MAP3K10** | | NM_002446 | **MFN2** | | NM_014874 |
| LOC391428 | XM_372953 | | **MAP3K11** | | NM_002419 | **MGC16169** | | NM_033115 |
| LOC391533 | XM_497921 | | **MAP3K12** | | NM_006301 | **MGC42105** | | NM_153361 |
| LOC392226 | XM_498286 | | **MAP3K13** | | NM_004721 | **MINK1** | | NM_015716 |
| LOC392265 | XM_498294 | | **MAP3K14** | | NM_003954 | **MINPP1** | | NM_004897 |
| LOC400301 | XM_375150 | | **MAP3K15** | | XM_372199 | **MKNK1** | | NM_003684 |
| LOC400708 | XM_375632 | | **MAP4K1** | | NM_007181 | **MKNK2** | | NM_017572 |
| LOC400927 | XM_376010 | | **MAP4K2** | | NM_004579 | **MLCK** | | NM_182493 |
| LOC402679 | XM_377958 | | **MAP4K3** | | NM_003618 | **MLH1** | | NM_000249 |
| LOC402679 | XM_380022 | | **MAP4K4** | | NM_145687 | **MLH3** | | NM_014381 |
| MLKL | NM_152649 | | **NLK** | | NM_016231 | **PDK1** | | NM_002610 |
| MLLT7 | NM_005938 | | **NME1** | | NM_000269 | **PDK2** | | NM_002611 |
| MOBK1B | NM_018221 | | **NME2** | | NM_002512 | **PDK4** | | NM_002612 |
| MOBKL1A | NM_173468 | | **NME3** | | NM_002513 | **PDPK1** | | NM_002613 |
| MOBKL2A | NM_130807 | | **NME4** | | NM_005009 | **PDXK** | | NM_003681 |
| MOBKL2B | NM_024761 | | **NME5** | | NM_003551 | **PFKFB1** | | NM_002625 |
| MORC1 | NM_014429 | | **NME7** | | NM_013330 | **PFKFB2** | | NM_006212 |
| MORC3 | NM_015358 | | **NPR2** | | NM_000907 | **PFKFB4** | | NM_004567 |
| MOS | NM_005372 | | **NR1H4** | | NM_005123 | **PFKL** | | NM_002626 |
| MPP1 | NM_002436 | | **NR1I2** | | NM_003889 | **PFKM** | | NM_000289 |
| MPP2 | NM_005374 | | **NR1I3** | | NM_005122 | **PFKP** | | NM_002627 |
| MPP3 | NM_001932 | | **NRAS** | | NM_002524 | **PFTK1** | | NM_012395 |
| MRE11A | NM_005591 | | **NRBP1** | | NM_013392 | **PGK1** | | NM_000291 |
| MSH2 | NM_000251 | | **NRBP2** | | NM_178564 | **PGK2** | | NM_138733 |
| MSH5 | NM_025259 | | **NRGN** | | NM_006176 | **PHACTR1** | | XM_166420 |
| MST1R | NM_002447 | | **NRK** | | NM_198465 | **PHACTR2** | | XM_376540 |
| MTM1 | NM_000252 | | **NTRK1** | | NM_002529 | **PHACTR3** | | NM_080672 |
| MTMR1 | NM_003828 | | **NTRK2** | | NM_006180 | **PHACTR4** | | NM_023923 |
| MTMR2 | NM_016156 | | **NTRK3** | | NM_002530 | **PHKA1** | | NM_002637 |
| MTMR3 | NM_021090 | | **NUAK1** | | NM_014840 | **PHKA2** | | NM_000292 |
| MTMR4 | NM_004687 | | **NUAK2** | | NM_030952 | **PHKB** | | NM_000293 |
| MTMR6 | NM_004685 | | **NUCKS1** | | NM_022731 | **PHKG1** | | NM_006213 |
| MTMR8 | NM_017677 | | **NUDT8** | | NM_181843 | **PHKG2** | | NM_000294 |
| MTMR9 | NM_015458 | | **OBSCN** | | NM_052843 | **PHLPP** | | NM_194449 |
| MTMR10 | NM_017762 | | **OTOF** | | NM_194323 | **PHLPPL** | | XM_041191 |
| MTMR12 | NM_019061 | | **OXSR1** | | NM_005109 | **PHOSPHO1** | | NM_178500 |
| MUSK | NM_005592 | | **P15RS** | | NM_018170 | **PI4K2B** | | NM_018323 |
| MVK | NM_000431 | | **PACSIN1** | | NM_020804 | **PI4KII** | | NM_018425 |
| MYB | NM_005375 | | **PACSIN2** | | NM_007229 | **PICK1** | | NM_012407 |
| MYC | NM_002467 | | **PACSIN3** | | NM_016223 | **PIK3AP1** | | NM_152309 |
| MYLK | NM_053028 | | **PAK1** | | NM_002576 | **PIK3C2A** | | NM_002645 |
| MYLK2 | NM_033118 | | **PAK2** | | NM_002577 | **PIK3C2B** | | NM_002646 |
| MYO3A | NM_017433 | | **PAK3** | | NM_002578 | **PIK3C2G** | | NM_004570 |
| MYO3B | NM_138995 | | **PAK4** | | NM_005884 | **PIK3C3** | | NM_002647 |
| MYO9B | NM_004145 | | **PAK6** | | NM_020168 | **PIK3CA** | | NM_006218 |
| MYST2 | NM_007067 | | **PAK7** | | NM_020341 | **PIK3CB** | | NM_006219 |
| NADK | NM_023018 | | **PANK1** | | NM_138316 | **PIK3CD** | | NM_005026 |
| NAGK | NM_017567 | | **PANK2** | | NM_024960 | **PIK3CG** | | NM_002649 |
| NBN | NM_002485 | | **PANK3** | | NM_024594 | **PIK3R1** | | NM_181504 |
| NEDD4L | NM_015277 | | **PANK4** | | NM_018216 | **PIK3R1** | | XM_043865 |
| NEK1 | NM_012224 | | **PAP2D** | | XM_375754 | **PIK3R2** | | NM_005027 |
| NEK2 | NM_002497 | | **PAPSS1** | | NM_005443 | **PIK3R3** | | NM_003629 |
| NEK3 | NM_152720 | | **PASK** | | NM_015148 | **PIK3R4** | | NM_014602 |
| NEK4 | NM_003157 | | **PBK** | | NM_018492 | **PIK3R5** | | NM_014308 |
| NEK5 | XM_292160 | | **PCK1** | | NM_002591 | **PIM1** | | NM_002648 |
| NEK6 | NM_014397 | | **PCK2** | | NM_004563 | **PIM2** | | NM_006875 |
| NEK7 | NM_133494 | | **PCTK1** | | NM_033018 | **PIM3** | | NM_001001852 |
| NEK8 | NM_178170 | | **PCTK2** | | NM_002595 | **PIN1** | | NM_006221 |
| NEK9 | NM_033116 | | **PCTK3** | | NM_002596 | **PINK1** | | NM_032409 |
| NEK10 | NM_152534 | | **PDGFB** | | NM_002608 | **PIP5K1A** | | NM_003557 |
| NEK11 | NM_024800 | | **PDGFRA** | | NM_006206 | **PIP5K1B** | | NM_003558 |
| NF1 | NM_000267 | | **PDGFRB** | | NM_002609 | **PIP5K1C** | | NM_012398 |
| NF2 | NM_000268 | | **PDGFRL** | | NM_006207 | **PIP5K2A** | | NM_005028 |
| NKX3-1 | NM_006167 | | **PDIK1L** | | NM_152835 | **PIP5K2B** | | NM_003559 |
| PIP5K2C | NM_024779 | | **PPM2C** | | NM_018444 | **PPP3R2** | | NM_147180 |
| PKIA | NM_006823 | | **PPME1** | | NM_016147 | **PPP4C** | | NM_002720 |
| PKIB | NM_032471 | | **PPP1CA** | | NM_002708 | **PPP4R1** | | NM_005134 |
| PKIG | NM_181805 | | **PPP1CB** | | NM_002709 | **PPP4R1L** | | XM_086650 |
| PKM2 | NM_182471 | | **PPP1CC** | | NM_002710 | **PPP4R2** | | NM_174907 |
| PKMYT1 | NM_004203 | | **PPP1R10** | | NM_002714 | **PPP5C** | | NM_006247 |
| PKN1 | NM_002741 | | **PPP1R11** | | NM_021959 | **PPP6C** | | NM_002721 |
| PKN2 | NM_006256 | | **PPP1R12A** | | NM_002480 | **PPTC7** | | NM_139283 |
| PLA2G4B | NM_005090 | | **PPP1R12B** | | NM_002481 | **PRKAA1** | | NM_006251 |
| PLAUR | NM_002659 | | **PPP1R12C** | | NM_017607 | **PRKAA2** | | NM_006252 |
| PLCB1 | NM_182734 | | **PPP1R13B** | | NM_015316 | **PRKAB2** | | NM_005399 |
| PLCB2 | NM_004573 | | **PPP1R14A** | | NM_033256 | **PRKACA** | | NM_002730 |
| PLCB3 | NM_000932 | | **PPP1R14B** | | XM_370630 | **PRKACB** | | NM_002731 |
| PLCB4 | NM_000933 | | **PPP1R14C** | | NM_030949 | **PRKAG1** | | NM_002733 |
| PLCD1 | NM_006225 | | **PPP1R14D** | | NM_017726 | **PRKAG2** | | NM_016203 |
| PLCD4 | NM_032726 | | **PPP1R15A** | | NM_014330 | **PRKAG3** | | NM_017431 |
| PLCG1 | NM_002660 | | **PPP1R15B** | | NM_032833 | **PRKAR1A** | | NM_002734 |
| PLCG2 | NM_002661 | | **PPP1R16A** | | NM_032902 | **PRKAR1B** | | NM_002735 |
| PLCL1 | NM_006226 | | **PPP1R16B** | | NM_015568 | **PRKAR2A** | | NM_004157 |
| PLCL2 | NM_015184 | | **PPP1R1A** | | NM_006741 | **PRKAR2B** | | NM_002736 |
| PLCZ1 | NM_033123 | | **PPP1R1B** | | NM_032192 | **PRKCA** | | NM_002737 |
| PLD1 | NM_002662 | | **PPP1R1C** | | XM_087137 | **PRKCB1** | | NM_002738 |
| PLK1 | NM_005030 | | **PPP1R2** | | NM_006241 | **PRKCBP1** | | NM_183048 |
| PLK2 | NM_006622 | | **PPP1R2P9** | | NM_025210 | **PRKCD** | | NM_006254 |
| PLK3 | NM_004073 | | **PPP1R3A** | | NM_002711 | **PRKCDBP** | | NM_145040 |
| PLK4 | NM_014264 | | **PPP1R3B** | | NM_024607 | **PRKCE** | | NM_005400 |
| PMS1 | NM_000534 | | **PPP1R3C** | | NM_005398 | **PRKCG** | | NM_002739 |
| PMVK | NM_006556 | | **PPP1R3D** | | NM_006242 | **PRKCH** | | NM_006255 |
| PNCK | NM_198452 | | **PPP1R3E** | | XM_033391 | **PRKCI** | | NM_002740 |
| POT1 | NM_015450 | | **PPP1R3F** | | XM_372210 | **PRKCQ** | | NM_006257 |
| PPAP2A | NM_003711 | | **PPP1R3G** | | XM_371796 | **PRKCSH** | | NM_001001329 |
| PPAP2C | NM_003712 | | **PPP1R7** | | NM_002712 | **PRKCZ** | | NM_002744 |
| PPAPDC1A | XM_113641 | | **PPP1R8** | | NM_002713 | **PRKD1** | | NM_002742 |
| PPAPDC2 | NM_203453 | | **PPP1R9A** | | XM_371933 | **PRKD2** | | NM_016457 |
| PPARA | NM_005036 | | **PPP1R9B** | | NM_032595 | **PRKD3** | | NM_005813 |
| PPARD | NM_006238 | | **PPP2CA** | | NM_002715 | **PRKDC** | | NM_006904 |
| Pparg | NM_011146 | | **PPP2CB** | | NM_004156 | **PRKG1** | | NM_006258 |
| PPARG | NM_138712 | | **PPP2R1A** | | NM_014225 | **PRKG2** | | NM_006259 |
| PPEF1 | NM_006240 | | **PPP2R1B** | | NM_002716 | **PRKRA** | | NM_003690 |
| PPEF2 | NM_006239 | | **PPP2R2A** | | NM_002717 | **PRKX** | | NM_005044 |
| PPFIA1 | NM_003626 | | **PPP2R2B** | | NM_004576 | **PRKY** | | NM_002760 |
| PPFIA2 | NM_003625 | | **PPP2R2C** | | NM_020416 | **PRPF4B** | | NM_003913 |
| PPFIA3 | NM_003660 | | **PPP2R2C** | | NM_181876 | **PRPS1** | | NM_002764 |
| PPFIA4 | XM_046751 | | **PPP2R3A** | | NM_002718 | **PRPS2** | | NM_002765 |
| PPFIBP1 | NM_003622 | | **PPP2R3B** | | NM_013239 | **PRSS7** | | NM_002772 |
| PPM1A | NM_021003 | | **PPP2R5A** | | NM_006243 | **PSKH1** | | NM_006742 |
| PPM1B | NM_002706 | | **PPP2R5B** | | NM_006244 | **PSKH2** | | NM_033126 |
| PPM1D | NM_003620 | | **PPP2R5C** | | NM_002719 | **PSMD14** | | NM_005805 |
| PPM1E | NM_014906 | | **PPP2R5D** | | NM_006245 | **PSPH** | | NM_004577 |
| PPM1F | NM_014634 | | **PPP2R5E** | | NM_006246 | **PSTPIP1** | | NM_003978 |
| PPM1H | XM_350880 | | **PPP3CA** | | NM_000944 | **PSTPIP2** | | NM_024430 |
| PPM1K | NM_152542 | | **PPP3CB** | | NM_021132 | **PTBP1** | | NM_002819 |
| PPM1L | NM_139245 | | **PPP3CC** | | NM_005605 | **PTCH** | | NM_000264 |
| PPM1M | NM_144641 | | **PPP3R1** | | NM_000945 | **PTCH2** | | NM_003738 |
| PTEN | NM_000314 | | **R3HDM2** | | NM_014925 | **RPS6KC1** | | NM_012424 |
| PTHR1 | NM_000316 | | **RAD50** | | NM_005732 | **RPS6KL1** | | NM_031464 |
| PTK2 | NM_005607 | | **RAF1** | | NM_002880 | **RSC1A1** | | NM_006511 |
| PTK2B | NM_004103 | | **RAGE** | | NM_014226 | **RXRA** | | NM_002957 |
| PTK6 | NM_005975 | | **RASA1** | | NM_022650 | **RXRB** | | NM_021976 |
| PTK7 | NM_002821 | | **RASA2** | | NM_006506 | **RXRG** | | NM_006917 |
| PTK9 | NM_002822 | | **RASA3** | | NM_007368 | **RYK** | | NM_002958 |
| PTK9L | NM_007284 | | **RASAL2** | | NM_004841 | **SAG** | | NM_000541 |
| PTN | NM_002825 | | **RASSF5** | | NM_031437 | **SBF1** | | NM_002972 |
| PTP4A1 | NM_003463 | | **RB1** | | NM_000321 | **SBF2** | | NM_030962 |
| PTP4A2 | NM_003479 | | **RBKS** | | NM_022128 | **SBK1** | | XM_370948 |
| PTP4A3 | NM_007079 | | **RBL1** | | NM_002895 | **SCAP1** | | NM_003726 |
| PTPDC1 | NM_152422 | | **RBL2** | | NM_005611 | **SCYL1** | | NM_020680 |
| PTPLA | NM_014241 | | **RCSD1** | | NM_052862 | **SCYL2** | | NM_017988 |
| PTPLAD2 | XM_376819 | | **REL** | | NM_002908 | **SCYL3** | | NM_020423 |
| PTPMT1 | XM_374879 | | **RET** | | NM_000323 | **SDHD** | | NM_003002 |
| PTPN1 | NM_002827 | | **RET** | | NM_020629 | **SETD2** | | NM_012271 |
| PTPN2 | NM_002828 | | **RFK** | | NM_018339 | **SF1** | | NM_004630 |
| PTPN3 | NM_002829 | | **RFP** | | NM_006510 | **SFN** | | NM_006142 |
| PTPN4 | NM_002830 | | **RGS3** | | NM_144489 | **SGK** | | NM_005627 |
| PTPN5 | NM_032781 | | **RHEB** | | NM_005614 | **SGK2** | | NM_170693 |
| PTPN6 | NM_002831 | | **RIC8B** | | NM_018157 | **SGK3** | | NM_013257 |
| PTPN7 | NM_002832 | | **RIMS1** | | NM_014989 | **SGK3** | | NM_170709 |
| PTPN9 | NM_002833 | | **RIMS4** | | NM_182970 | **SH2D1A** | | NM_002351 |
| PTPN12 | NM_002835 | | **RIOK1** | | NM_031480 | **SH2D1B** | | NM_053282 |
| PTPN13 | NM_006264 | | **RIOK2** | | NM_018343 | **SH3KBP1** | | NM_031892 |
| PTPN14 | NM_005401 | | **RIPK1** | | NM_003804 | **SHC1** | | NM_003029 |
| PTPN18 | NM_014369 | | **RIPK2** | | NM_003821 | **SHH** | | NM_000193 |
| PTPN21 | NM_007039 | | **RIPK3** | | NM_006871 | **SIRPA** | | NM_080792 |
| PTPN22 | NM_012411 | | **RIPK4** | | NM_020639 | **SIRPB2** | | XM_209363 |
| PTPN23 | NM_015466 | | **RIPK5** | | NM_015375 | **SIRPD** | | NM_178460 |
| PTPRA | NM_002836 | | **RNASEL** | | NM_021133 | **SIRT2** | | NM_012237 |
| PTPRB | NM_002837 | | **RNF180** | | NM_178532 | **SKI** | | NM_003036 |
| PTPRC | NM_002838 | | **RNGTT** | | NM_003800 | **SKIP** | | XM_051221 |
| PTPRCAP | NM_005608 | | **ROCK1** | | NM_005406 | **SKIP** | | NM_016532 |
| PTPRD | NM_002839 | | **ROCK2** | | NM_004850 | **Slc1a3** | | NM_148938 |
| PTPRE | NM_006504 | | **ROR1** | | NM_005012 | **SLC22A18** | | NM_002555 |
| PTPRF | NM_002840 | | **ROR2** | | NM_004560 | **Slc26a9** | | NM_177243 |
| PTPRG | NM_002841 | | **ROS1** | | NM_002944 | **SLK** | | NM_014720 |
| PTPRH | NM_002842 | | **RP11-145H9.1** | | XM_373109 | **SMAD2** | | NM_005901 |
| PTPRJ | NM_002843 | | **RP6-213H19.1** | | NM_016542 | **SMAD4** | | NM_005359 |
| PTPRK | NM_002844 | | **RPA1** | | NM_002945 | **SMARCB1** | | NM_003073 |
| PTPRM | NM_002845 | | **RPA2** | | NM_002946 | **SMG1** | | NM_014006 |
| PTPRN | NM_002846 | | **RPGRIP1** | | NM_020366 | **SMG6** | | NM_017575 |
| PTPRN2 | NM_002847 | | **RPH3A** | | NM_014954 | **SNAI3** | | XM_370995 |
| PTPRO | NM_002848 | | **RPS6** | | NM_001010 | **SNF1LK** | | NM_173354 |
| PTPRR | NM_002849 | | **RPS6KA1** | | NM_002953 | **SNF1LK2** | | NM_015191 |
| PTPRS | NM_002850 | | **RPS6KA2** | | NM_021135 | **SNRK** | | NM_017719 |
| PTPRT | NM_007050 | | **RPS6KA3** | | NM_004586 | **SOCS5** | | NM_014011 |
| PTPRU | NM_005704 | | **RPS6KA4** | | NM_003942 | **SOD1** | | NM_000454 |
| PTPRV | XM_086287 | | **RPS6KA5** | | NM_004755 | **SOX2** | | NM_003106 |
| PTPRZ1 | NM_002851 | | **RPS6KA6** | | NM_014496 | **SPEG** | | NM_005876 |
| PXK | NM_017771 | | **RPS6KB1** | | NM_003161 | **SRMS** | | NM_080823 |
| R3HDM1 | NM_015361 | | **RPS6KB2** | | NM_003952 | **SRPK2** | | NM_003138 |
| SSH1 | NM_018984 | | **TESK1** | | NM_006285 | **TYROBP** | | NM_003332 |
| SSH2 | NM_033389 | | **TESK2** | | NM_007170 | **UCK1** | | NM_031432 |
| SSH3 | NM_017857 | | **TEX14** | | NM_031272 | **UCK2** | | NM_012474 |
| STAC3 | NM_145064 | | **TGFA** | | NM_003236 | **UCKL1** | | NM_017859 |
| STK3 | NM_006281 | | **TGFBR1** | | NM_004612 | **UGP2** | | NM_006759 |
| STK4 | NM_006282 | | **TGFBR2** | | NM_003242 | **UHMK1** | | NM_144624 |
| STK10 | NM_005990 | | **THBS1** | | NM_003246 | **ULK1** | | NM_003565 |
| STK11 | NM_000455 | | **THOC4** | | NM_005782 | **ULK2** | | NM_014683 |
| STK11IP | NM_052902 | | **TIAM1** | | NM_003253 | **ULK3** | | NM_015518 |
| STK16 | NM_003691 | | **TIE1** | | NM_005424 | **ULK4** | | NM_017886 |
| STK17A | NM_004760 | | **TINF2** | | NM_012461 | **UNC13B** | | NM_006377 |
| STK17B | NM_004226 | | **TJP2** | | NM_004817 | **UNK** | | XM_062966 |
| STK19 | NM_004197 | | **TK1** | | NM_003258 | **UNK** | | XM_171165 |
| STK23 | NM_014370 | | **TK2** | | NM_004614 | **UNK** | | XM_291584 |
| STK24 | NM_003576 | | **TLK1** | | NM_012290 | **UNK** | | XM_291786 |
| STK25 | NM_006374 | | **TLK2** | | NM_006852 | **UNK** | | XM_370946 |
| STK31 | NM_032944 | | **TNFRSF11B** | | NM_002546 | **UNK** | | XM_371492 |
| STK32A | NM_145001 | | **TNIK** | | XM_039796 | **UNK** | | XM_372542 |
| STK32B | NM_018401 | | **TNK1** | | NM_003985 | **UNK** | | XM_372625 |
| STK32C | NM_173575 | | **TNK2** | | NM_005781 | **UNK** | | XM_372987 |
| STK33 | NM_030906 | | **TNKS** | | NM_003747 | **UNK** | | XM_373224 |
| STK35 | NM_080836 | | **TNNI3K** | | NM_015978 | **UNK** | | XM_373298 |
| STK36 | NM_015690 | | **TNS1** | | NM_022648 | **UNK** | | XM_373815 |
| STK38 | NM_007271 | | **TNS3** | | NM_022748 | **UNK** | | XM_376585 |
| STK38L | NM_015000 | | **TP53RK** | | NM_033550 | **UNK** | | XM_376950 |
| STK39 | NM_013233 | | **TPD52L3** | | NM_033516 | **UNK** | | XM_377635 |
| STK40 | NM_032017 | | **TPK1** | | NM_022445 | **UNK** | | XM_378103 |
| STYK1 | NM_018423 | | **TPTE2** | | NM_130785 | **UNK** | | XM_378155 |
| STYX | NM_145251 | | **TPTEps1** | | XM_495953 | **UNK** | | XM_378664 |
| STYXL1 | NM_016086 | | **TRAF3IP3** | | NM_025228 | **UNK** | | XM_495804 |
| SUV39H2 | NM_024670 | | **TRIB1** | | NM_025195 | **UNK** | | XM_496486 |
| SUZ12 | NM_015355 | | **TRIB2** | | NM_021643 | **UNK** | | XM_496630 |
| SYK | NM_003177 | | **TRIB3** | | NM_021158 | **UNK** | | XM_496720 |
| SYT2 | NM_177402 | | **TRIO** | | NM_007118 | **UNK** | | XM_496793 |
| SYT4 | NM_020783 | | **TRPM6** | | NM_017662 | **UNK** | | XM_496862 |
| SYT5 | NM_003180 | | **TRPM7** | | NM_017672 | **UNK** | | XM_497237 |
| SYT11 | NM_152280 | | **TRPV5** | | NM_019841 | **UNK** | | XM_497414 |
| SYT14 | NM_153262 | | **TRPV6** | | NM_018646 | **UNK** | | XM_497433 |
| SYT16 | NM_031914 | | **TSC1** | | NM_000368 | **UNK** | | XM_497521 |
| SYT17 | NM_016524 | | **TSC2** | | NM_000548 | **UNK** | | XM_497706 |
| SYTL5 | NM_138780 | | **TSKS** | | NM_021733 | **UNK** | | XM_497790 |
| TAF1 | NM_004606 | | **TSSK1** | | NM_032028 | **UNK** | | XM_497791 |
| TAF1L | NM_153809 | | **TSSK2** | | NM_053006 | **UNK** | | XM_497812 |
| TAOK1 | NM_020791 | | **TSSK3** | | NM_052841 | **UNK** | | XM_497846 |
| TAOK2 | NM_016151 | | **TSSK4** | | NM_174944 | **UNK** | | XM_497909 |
| TAOK2 | NM_004783 | | **TSSK6** | | NM_032037 | **UNK** | | XM_498204 |
| TAOK3 | NM_016281 | | **TTBK1** | | XM_166453 | **UNK** | | XM_498243 |
| TBK1 | NM_013254 | | **TTBK2** | | NM_173500 | **UNK** | | XM_498259 |
| TEC | NM_003215 | | **TTK** | | NM_003318 | **UNK** | | XM_498262 |
| TEK | NM_000459 | | **TTN** | | NM_003319 | **UNK** | | XM_499394 |
| TENC1 | NM_170754 | | **TTRAP** | | NM_016614 | **UNK** | | XM_499479 |
| TEP1 | NM_007110 | | **TXK** | | NM_003328 | **VAV1** | | NM_005428 |
| TERF1 | NM_017489 | | **TYK2** | | NM_003331 | **VHL** | | NM_000551 |
| TERF2IP | NM_018975 | | **TYRO3** | | NM_006293 | **VRK1** | | NM_003384 |
| VRK2 | NM_006296 | | **WNK4** | | NM_032387 | **XRCC6** | | NM_001469 |
| VRK3 | NM_016440 | | **WNT1** | | NM_005430 | **XYLB** | | NM_005108 |
| WEE1 | NM_003390 | | **WT1** | | NM_024426 | **YES1** | | NM_005433 |
| WIF1 | NM_007191 | | **WTAP** | | NM_004906 | **YSK4** | | NM_025052 |
| WNK1 | NM_018979 | | **WWP2** | | NM_007014 | **ZAK** | | NM_016653 |
| WNK2 | NM_006648 | | **XRCC4** | | NM_022406 | **ZAP70** | | NM_001079 |
| WNK3 | NM_020922 | | **XRCC5** | | NM_021141 | **ZC3HC1** | | NM_016478 |
