# Supplementary material for: shRNA-Based Screen Identifies Endocytic Recycling Pathway Components That Act as Genetic Modifiers of Alpha-Synuclein Aggregation, Secretion and Toxicity
Source: PLoS Genet. 2016 Apr 28;12(4):e1005995. doi: 10.1371/journal.pgen.1005995 (PMC4849646; doi:10.1371/journal.pgen.1005995)
Supplement: S2 Table — (DOCX) [file pgen.1005995.s002.docx]

**S2 Table. Summary of the effect of traffic players on oligomerization and aggregation of aSyn**

|  | **aSyn-BiFC system** | | | | | | | | | | **aSyn aggregation** | | | | | | | | | | | |
| --- | --- | --- | --- | --- | --- | --- | --- | --- | --- | --- | --- | --- | --- | --- | --- | --- | --- | --- | --- | --- | --- | --- |
|  | **Fluorescence  intensity** | | **aSyn  protein levels** | | **Cell-to-cell traffic** | **Secretion** | **Cytotoxicity** | | **Tranferrin  intensity** | | **number of inclusions per cell** | | | | | | **Secretion** | | **Cytotoxicity** | | **Transferrin intensity** | |
|  |  |  |  |  |  |  |  |  |  |  | no inclusions | | <10 | | >10 | |  |  |  |  |  |  |
|  | **KD^[[1]](#footnote-1)^** | **OE^[[2]](#footnote-2)^** | **KD** | **OE** | **KD** | **OE** | **KD** | **OE** | **KD** | **OE** | **KD** | **OE** | **KD** | **OE** | **KD** | **OE** | **KD** | **OE** | **KD** | **OE** | **KD** | **OE** |
| ***RAB8B*** | **↑** | **↓** | **↑** | **↔** | **↑** | **↔** | **↑** | **↓** | **−** | **↑** | **↓** | **↑** | **↓** | **↓** | **↑** | **↔** | **−** | **↔** | **↑** | **↓** | **−** | **↓** |
| ***RAB11A*** | **↑** | **↓** | **↔** | **↔** | **↔** | **↔** | **↔** | **↓** | **−** | **↓** | **↓** | **↑** | **↑** | **↓** | **↔** | **↔** | **−** | **↑** | **↔** | **↓** | **−** | **↓** |
| ***RAB13*** | **↑** | **↓** | **↓** | **↔** | **↑** | **↔** | **↑** | **↓** | **−** | **↓** | **↓** | **↑** | **↔** | **↓** | **↑** | **↔** | **−** | **↑** | **↔** | **↓** | **−** | **↓** |
| ***RAB39B*** | **↑** | **−** | **↔** | **−** | **−** | **−** | **↔** | **−** | **−** | **−** | **↓** | **−** | **↑** | **−** | **↔** | **−** | **−** | **−** | **↑** | **−** | **−** | **−** |
| **RAB27A** | **↑** | **−** | **↔** | **−** | **↔** | **−** | **↔** |  | **−** | **−** | **↓** | **−** | **↓** | **−** | **↑** | **−** | **−** | **−** | **↔** | **−** | **−** | **−** |
| ***CAMK1*** | **↑** | **−** | **↑** | **−** | **−** | **−** | **↔** | **−** | **−** | **−** | **↓** | **−** | **↑** | **−** | **↔** | **−** | **−** | **−** | **↔** | **−** | **−** | **−** |
| ***DYRK2*** | **↑** | **−** | **↔** | **−** | **−** | **−** | **↔** | **−** | **−** | **−** | **↑** | **−** | **↓** | **−** | **↔** | **−** | **−** | **−** | **↔** | **−** | **−** | **−** |
| ***CC2D1A*** | **↓** | **−** | **↓** | **−** | **−** | **−** | **↔** | **−** | **−** | **−** | **↓** | **−** | **↔** | **−** | **↑** | **−** | **−** | **−** | **↔** | **−** | **−** | **−** |
| ***CLK4*** | **↓** | **−** | **↓** | **−** | **−** | **−** | **↑** | **−** | **−** | **−** | **↔** | **−** | **↓** | **−** | **↑** | **−** | **−** | **−** | **↔** | **−** | **−** | **−** |
| ***SYTL5*** | **↓** | **↔** | **↓** | **↔** | **↑** | **↑** | **↔** | **↓** | **−** | **↔** | **↓** | **↑** | **↓** | **↓** | **↑** | **↔** | **−** | **↔** | **↔** | **↓** | **−** | **↓** |

1. KD, knockdown [↑](#footnote-ref-1)
2. OE, overexpression [↑](#footnote-ref-2)
