# Supplementary figures and images for: shRNA-Based Screen Identifies Endocytic Recycling Pathway Components That Act as Genetic Modifiers of Alpha-Synuclein Aggregation, Secretion and Toxicity
Source: PLoS Genet. 2016 Apr 28;12(4):e1005995. doi: 10.1371/journal.pgen.1005995 (PMC4849646; doi:10.1371/journal.pgen.1005995)

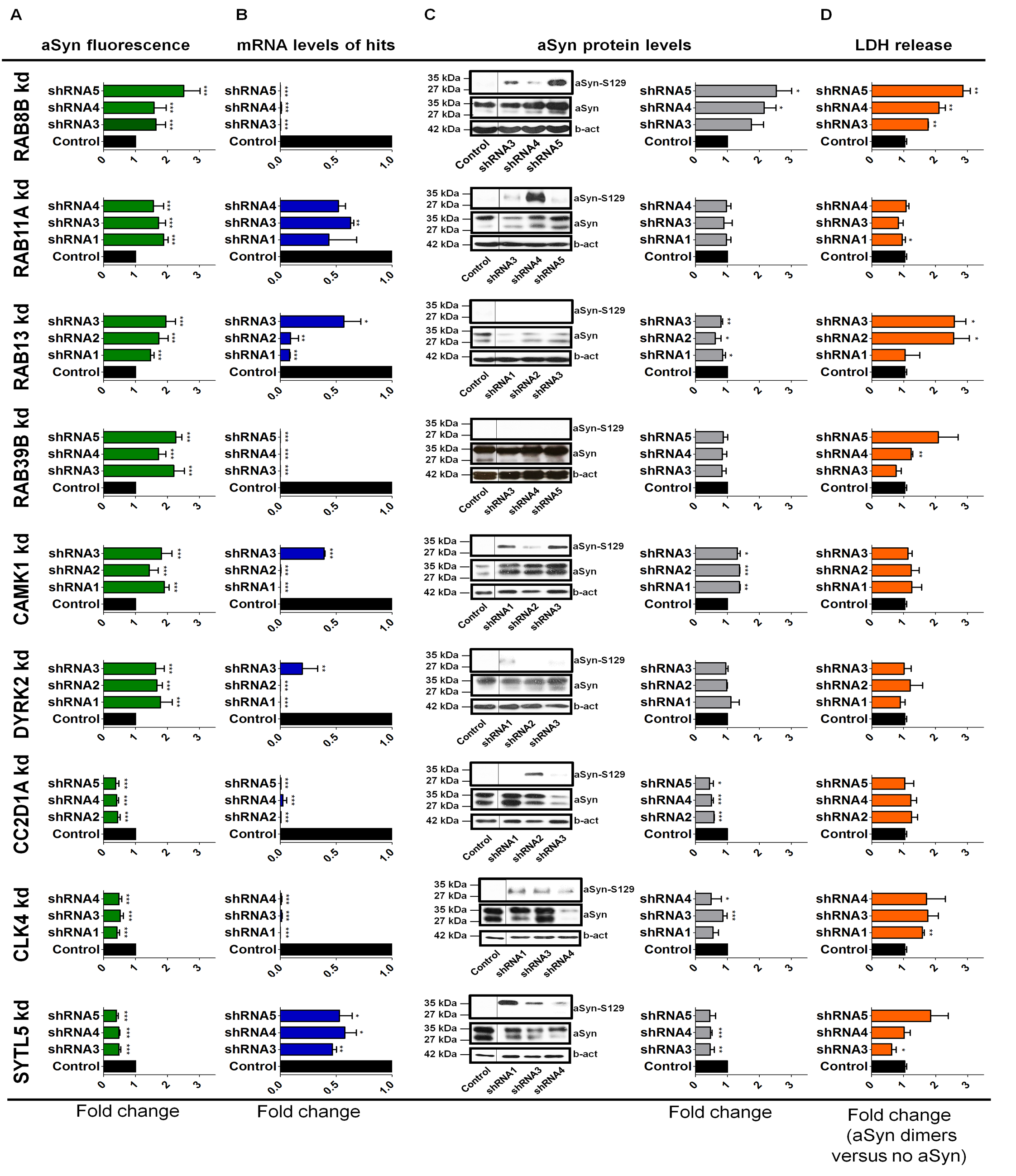

Supplement: S1 Fig — A. Quantification of relative fluorescence intensity of aSyn-BiFC stable H4 cells submitted to silencing of RAB8B, RAB11A, RAB13, RAB39B, CAMK1 DYRK2, CC2D1A, CLK4 and SYTL5. Three different shRNAs were used per gene. B. mRNA levels of cells submitted to silencing of the hits normalized to control cells. C. Immunoblotting analysis of S129 phosphorylated aSyn, total aSyn and beta-actin. Quantification of aSyn protein levels from aSyn-BiFC cells submitted to silencing of the selected hits D. Cytotoxicity (measured by LDH release in media from cells with aSyn oligomers versus no aSyn) normalized to control cells. All the quantifications presented are normalized to the control cells infected with a scrambled shRNA. Bars represent mean±95% CI (*: 0.050.01; **: 0.010.001; ***: p<0.001) and are normalized to the control of at least three independent experiments. Single comparisons between the control and experimental groups were made through Wilcoxon test. kd, knockdown. (TIF) [file pgen.1005995.s003.tif]

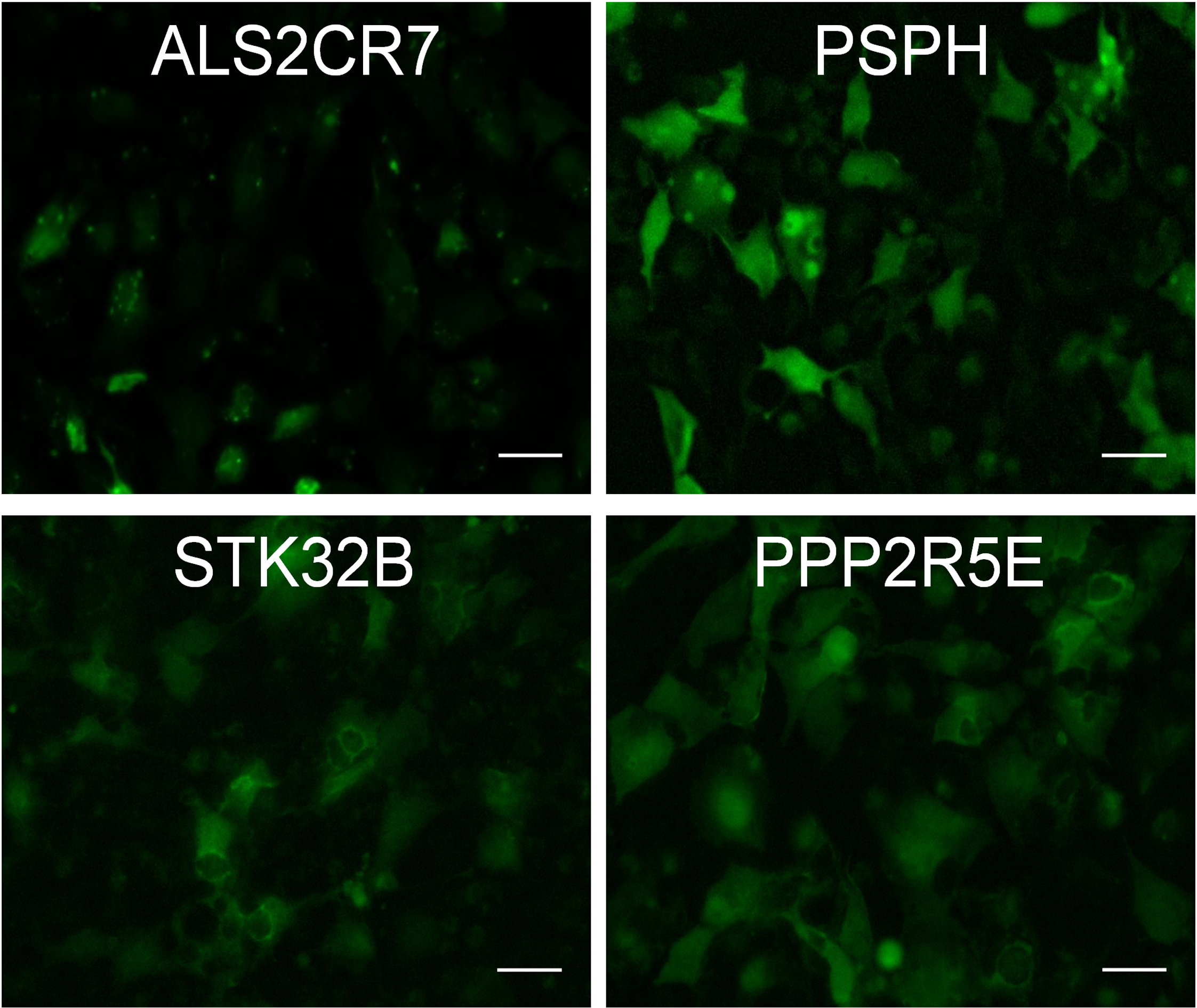

Supplement: S2 Fig — Upon silencing of ALS2CR7 or PSPH, aSyn aggregates are seen within cells. Silencing of STK32B and PPP2R5E leads to reduced fluorescence in the nucleus. In addition, a ring of fluorescent signal surrounding the nucleus is observed. Scale bars: 20 μm. (TIF) [file pgen.1005995.s004.tif]

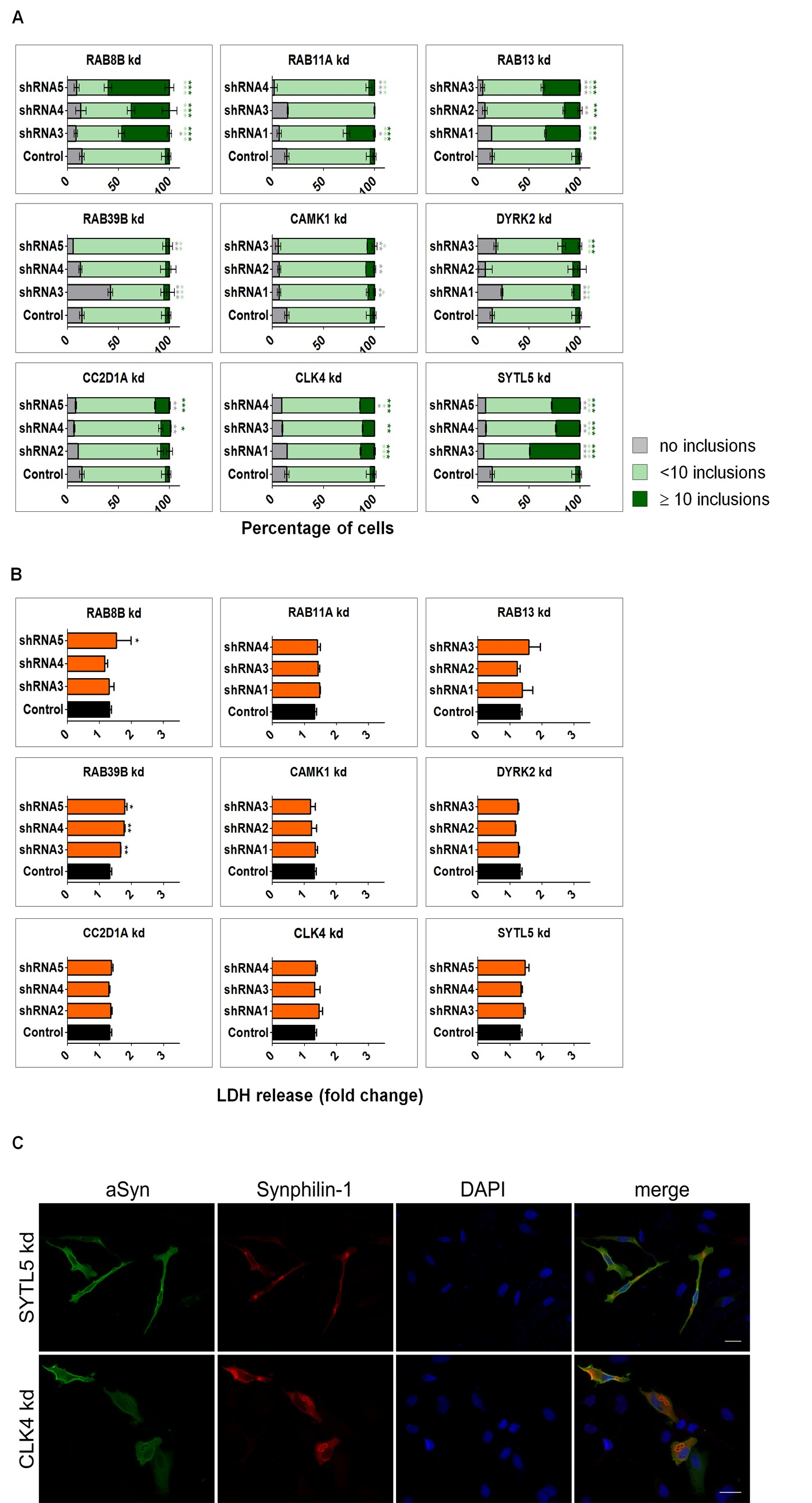

Supplement: S3 Fig — A. Quantification of the number of aSyn inclusions per cell. 3 different shRNAs per gene were used. The number of inclusions was divided in the following categories: no inclusions (gray), less than 10 inclusions (light green) and more than 10 inclusions (dark green). B. Cytotoxicity (measured by LDH release in media) from cells with aSyn inclusions versus no aSyn and normalized to control cells. All quantifications are normalized to the control (scrambled infected cells). Bars represent mean±95% CI (*: 0.050.01; **: 0.010.001; ***: p<0.001) and are normalized to the control of at least three independent experiments. Single comparisons between the control and experimental groups were made through Wilcoxon test. C. Immunohistochemistry of cells expressing aSyn and silenced for CLK4 and SYTL5. Silencing of SYTL5 in aSyn-expressing cells promote cell elongation. Upon CLK4 depletion, aSyn inclusions adopt an amorphous shape. Scale bars: 20 μm. kd, knockdown. (TIF) [file pgen.1005995.s005.tif]

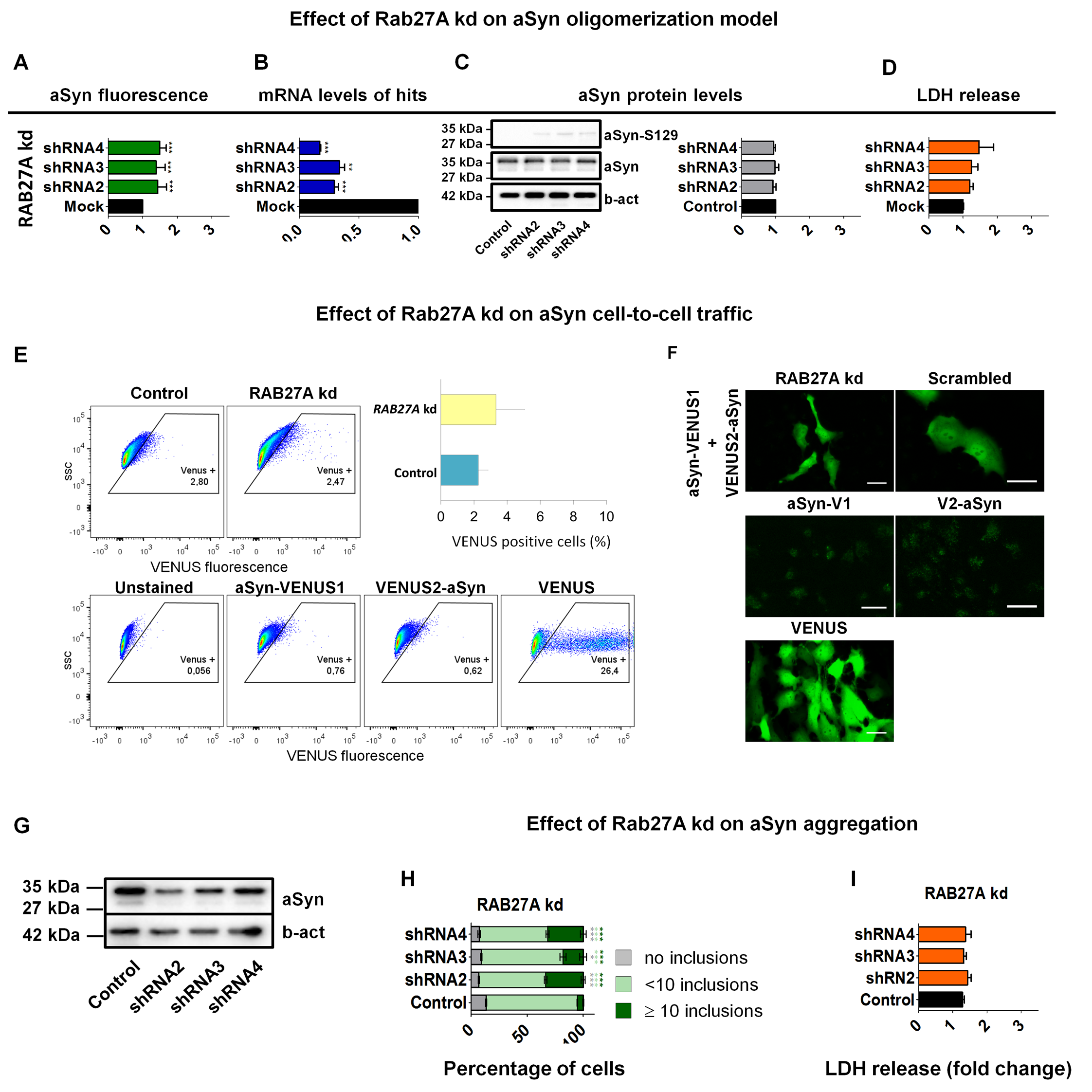

Supplement: S4 Fig — A. Quantification of relative fluorescence intensity of aSyn-BiFC stable H4 cells submitted to silencing of RA27A. Three different shRNAs were tested. B. mRNA levels of cells submitted to silencing of the RAB27A normalized to control cells (cells transduced with scrambled shRNA). C. Immunoblotting analysis of S129 phosphorylated aSyn, total aSyn and beta-actin. Quantification of aSyn protein levels from aSyn-BiFC cells submitted to silencing of RAB27A D. Cytotoxicity (measured by LDH release in media from cells with aSyn oligomers versus no aSyn) normalized to control cells. E. VENUS positive cells were monitored by flow cytometry. A representative result is shown as side scatter (SSC) versus VENUS fluorescence, with the corresponding histogram. F. In vivo imaging of aSyn-VENUS1 and VENUS2-aSyn mixed cells subjected to silencing of RAB27A. Scale bar: 20 μm. G. Immunoblotting analysis of total aSyn and beta-actin. H. Percentage of cells with no inclusions (gray), less than 10 inclusions (light green) or more than 10 inclusions (dark green). I. Cytotoxicity (measured by LDH release in the media) from stable cells subjected to RAB27A silencing and normalized to control. Bars represent mean±95% CI (*: 0.050.01; **: 0.010.001; ***: p<0.001) and are normalized to the control of at least three independent experiments. Single comparisons between the control and experimental groups were made Wilcoxon test. kd, knockdown. (TIF) [file pgen.1005995.s006.tif]

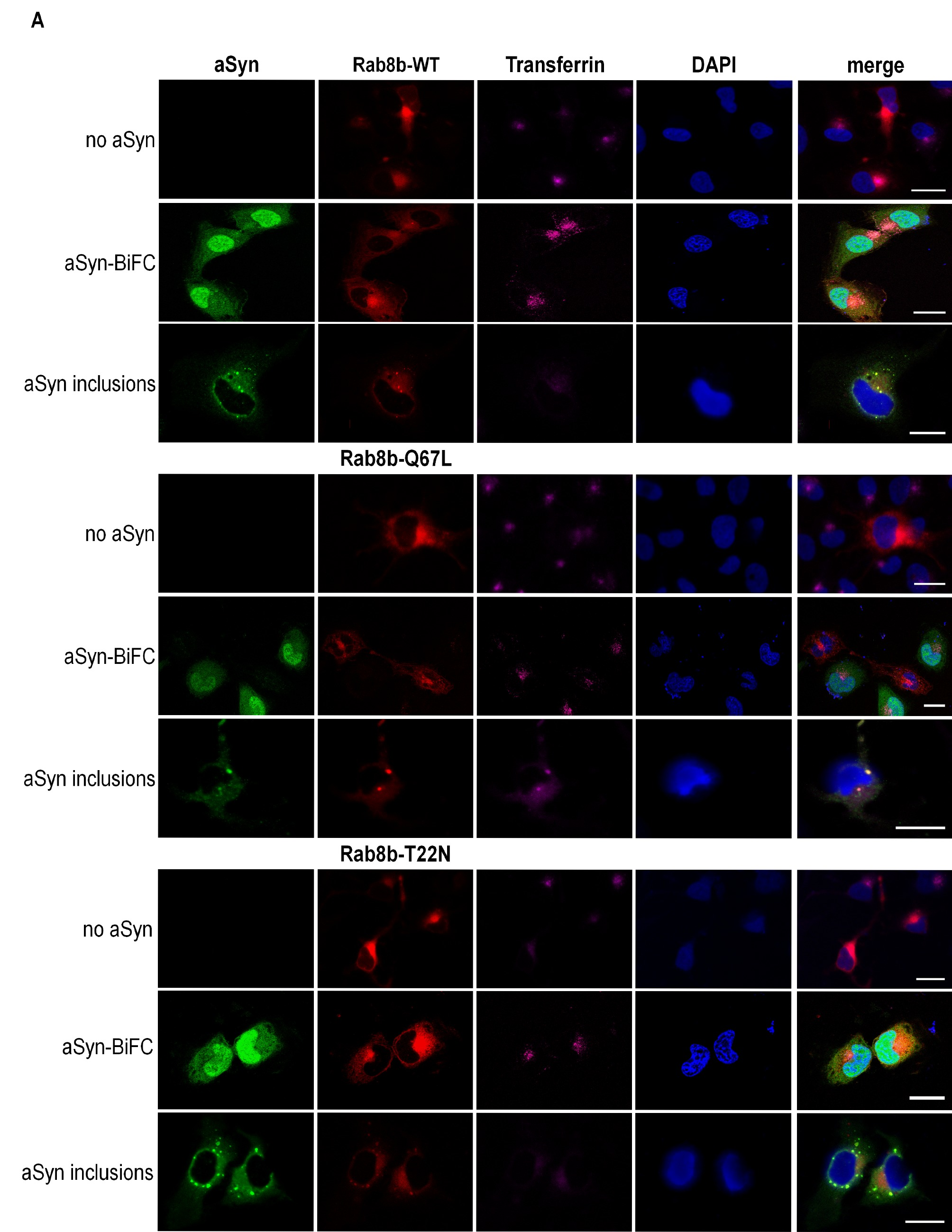

Supplement: S5 Fig — H4 cells with no aSyn or stable for aSyn-BiFC (green) were transfected with Rab8b-WT,–Q67L and–T22N constructs. To promote the formation of aSyn inclusions, cells were triple-transfected with aSynT, Synphilin-1 and the same constructs referred above. 48 h post-transfection, media with no serum was replaced in cells for 1 h. Cells were incubated with Alexa-647 human transferrin (magenta) for 30 min, prior to fixation. DAPI was used as a nuclear counterstain. Only for aSyn aggregation model, cells were subjected to immunocytochemistry for aSyn (green) followed by confocal microscopy. Scale bars: 20 μm. Control cells are represented in S8B Fig. (TIF) [file pgen.1005995.s007.tif]

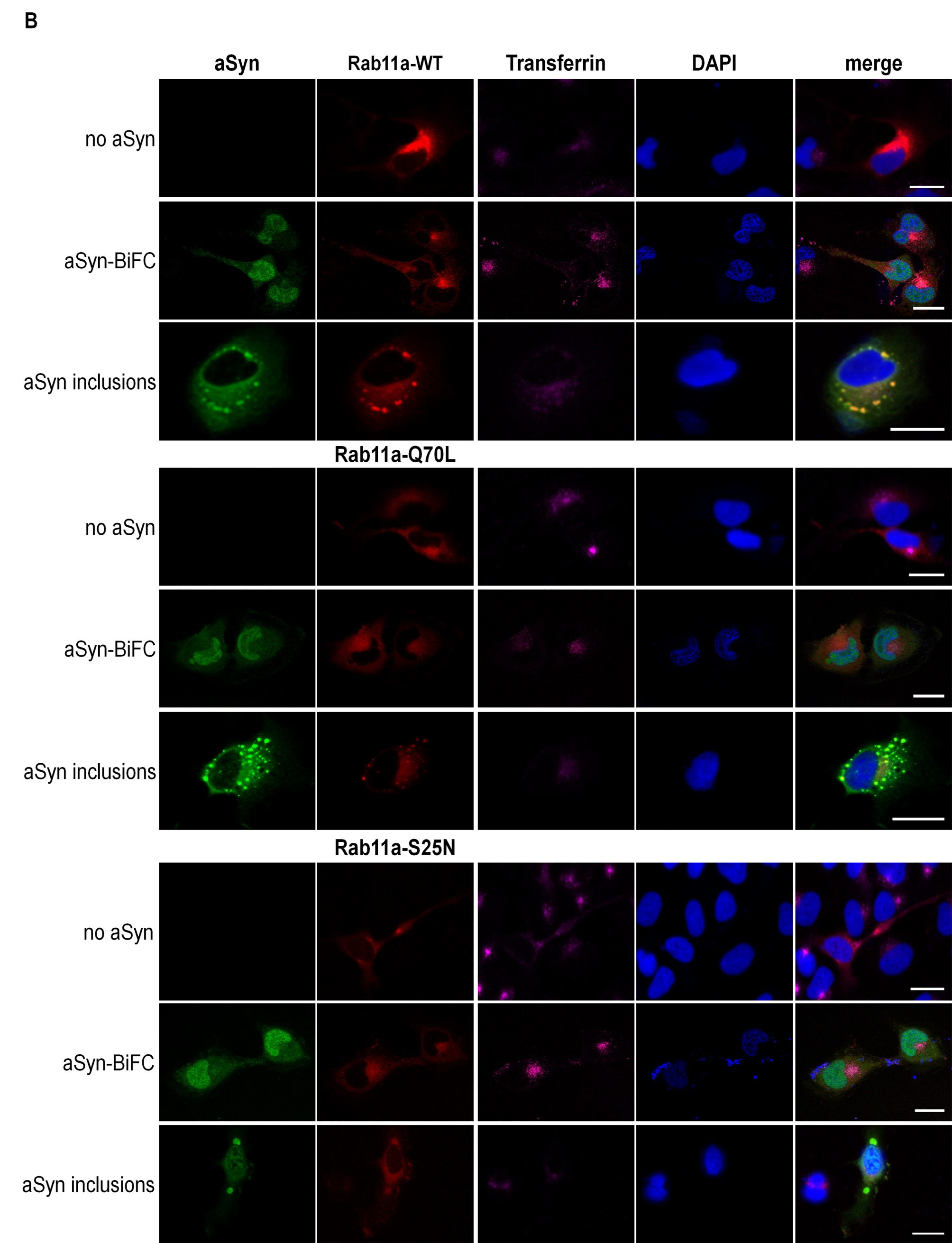

Supplement: S6 Fig — H4 cells with no aSyn or stable for aSyn-BiFC (green) were transfected with constructs expressing Rab11a-WT, Q70L and -S25N. To promote the formation of aSyn inclusions, cells were triple-transfected with aSynT, Synphilin-1 and the same constructs referred above. 48 h post-transfection, media with no serum was replaced in cells for 1 h. Cells were incubated with Alexa-647 human transferrin (magenta) for 30 min, prior to fixation. DAPI was used as a nuclear counterstain. Only for aSyn aggregation model, cells were subjected to immunocytochemistry for aSyn (green) followed by confocal microscopy. Scale bars: 20 μm. Control cells are represented in S8B Fig. (TIF) [file pgen.1005995.s008.tif]

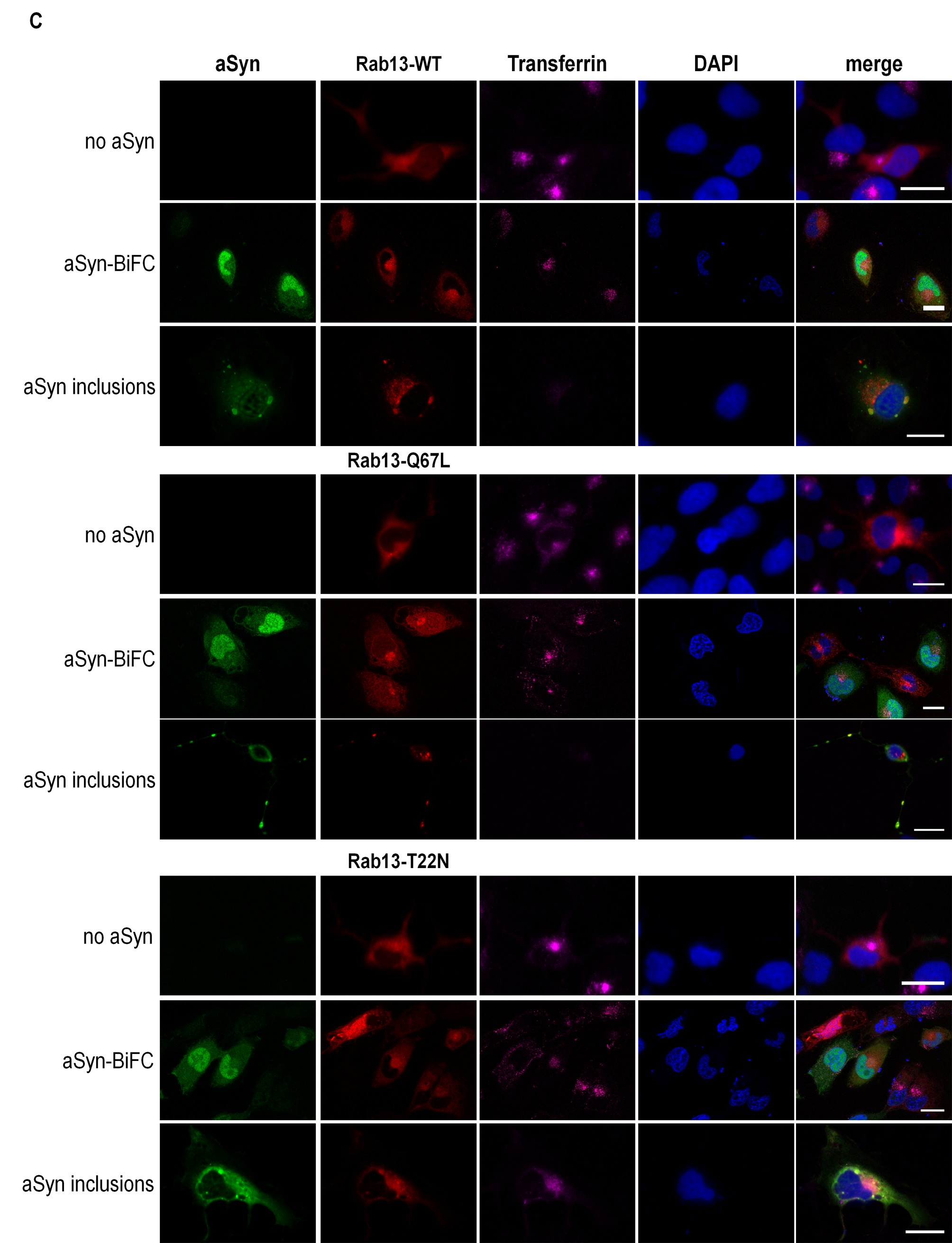

Supplement: S7 Fig — H4 cells with no aSyn or stable for aSyn-BiFC (green) were transfected with constructs expressing Rab13-WT, –67L and–T22N. To promote the formation of aSyn inclusions, cells were triple-transfected with aSynT, Synphilin-1 and the same constructs referred above. 48 h post-transfection, media with no serum was replaced in cells for 1 h. Cells were incubated with Alexa-647 human transferrin (magenta) for 30 min, prior to fixation. DAPI was used as a nuclear counterstain. Only for aSyn aggregation model, cells were subjected to immunocytochemistry for aSyn (green) followed by confocal microscopy. Scale bars: 20 μm. Control cells are represented in S8B Fig. (TIF) [file pgen.1005995.s009.tif]

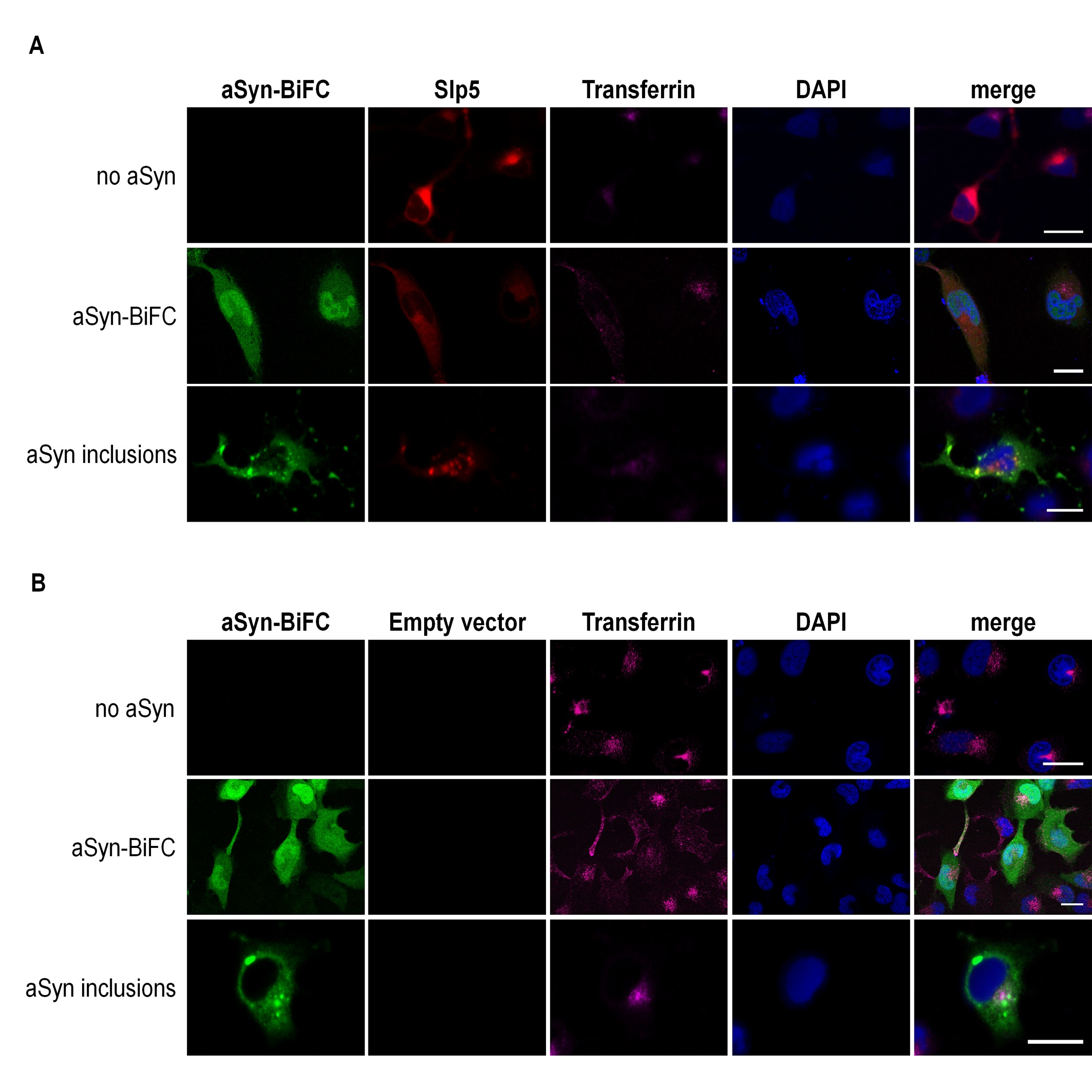

Supplement: S8 Fig — H4 cells with no aSyn or stable for aSyn-BiFC (green) were transfected with (A) SLP5 or (B) empty vector. To promote the formation of aSyn inclusions, cells were triple-transfected with aSynT, Synphilin-1 and the same constructs referred above. 48 h post-transfection, media with no serum was replaced in cells for 1 h. Cells were incubated with Alexa-647 human transferrin (magenta) for 30 min, prior to fixation. DAPI was used as a nuclear counterstain. Only for aSyn aggregation model, cells were subjected to immunocytochemistry for aSyn (green) followed by confocal microscopy. Scale bars: 20 μm. (TIF) [file pgen.1005995.s010.tif]
